# Mass Spectrometry-Based Proteomics Reveal Alcohol Dehydrogenase 1B as a Blood Biomarker Candidate to Monitor Acetaminophen-Induced Liver Injury

**DOI:** 10.3390/ijms222011071

**Published:** 2021-10-14

**Authors:** Floriane Pailleux, Pauline Maes, Michel Jaquinod, Justine Barthelon, Marion Darnaud, Claire Lacoste, Yves Vandenbrouck, Benoît Gilquin, Mathilde Louwagie, Anne-Marie Hesse, Alexandra Kraut, Jérôme Garin, Vincent Leroy, Jean-Pierre Zarski, Christophe Bruley, Yohann Couté, Didier Samuel, Philippe Ichai, Jamila Faivre, Virginie Brun

**Affiliations:** 1 Univ. Grenoble Alpes, INSERM, CEA, UMR BioSanté U1292, CNRS, CEA, FR2048, 38000 Grenoble, France; floriane.pailleux@yahoo.fr (F.P.); pauline.maes1001@gmail.com (P.M.); michel.jaquinod@cea.fr (M.J.); justine.barthelon@gmail.com (J.B.); yves.vandenbrouck@cea.fr (Y.V.); benoit.gilquin@cea.fr (B.G.); mathilde.louwagie@cea.fr (M.L.); anne-marie.hesse@cea.fr (A.-M.H.); alexandra.kraut@cea.fr (A.K.); jerome.garin@cea.fr (J.G.); christophe.bruley@cea.fr (C.B.); yohann.coute@cea.fr (Y.C.); 2 Clinique Universitaire d’Hépato-gastroentérologie, Centre Hospitalier Universitaire Grenoble, 38000 Grenoble, France; vincent.leroy@hmn.aphp.fr (V.L.); jpzarski@chu-grenoble.fr (J.-P.Z.); 3 Hepatobiliary Centre, Paul-Brousse University Hospital, INSERM U1193, 94800 Villejuif, France; marion.darnaud@bioaster.org (M.D.); clacostefr@yahoo.fr (C.L.); didier.samuel@aphp.fr (D.S.); philippe.ichai@aphp.fr (P.I.); 4 Faculté de Médecine, Université Paris-Sud, Université Paris-Saclay, 94270 Le Kremlin-Bicêtre, France; 5 Univ. Grenoble Alpes, CEA, LETI, Clinatec, 38000 Grenoble, France; 6 Institute for Advanced Biosciences, Université Grenoble Alpes, CNRS, INSERM U1209, 38000 Grenoble, France; 7 Assistance Publique-Hôpitaux de Paris (AP-HP), Pôle de Biologie Médicale, Paul-Brousse University Hospital, 94800 Villejuif, France

**Keywords:** bioinformatics, proteomics, mass spectrometry, liver, blood, biomarker, acetaminophen

## Abstract

Acute liver injury (ALI) is a severe disorder resulting from excessive hepatocyte cell death, and frequently caused by acetaminophen intoxication. Clinical management of ALI progression is hampered by the dearth of blood biomarkers available. In this study, a bioinformatics workflow was developed to screen omics databases and identify potential biomarkers for hepatocyte cell death. Then, discovery proteomics was harnessed to select from among these candidates those that were specifically detected in the blood of acetaminophen-induced ALI patients. Among these candidates, the isoenzyme alcohol dehydrogenase 1B (ADH1B) was massively leaked into the blood. To evaluate ADH1B, we developed a targeted proteomics assay and quantified ADH1B in serum samples collected at different times from 17 patients admitted for acetaminophen-induced ALI. Serum ADH1B concentrations increased markedly during the acute phase of the disease, and dropped to undetectable levels during recovery. In contrast to alanine aminotransferase activity, the rapid drop in circulating ADH1B concentrations was followed by an improvement in the international normalized ratio (INR) within 10–48 h, and was associated with favorable outcomes. In conclusion, the combination of omics data exploration and proteomics revealed ADH1B as a new blood biomarker candidate that could be useful for the monitoring of acetaminophen-induced ALI.

## 1. Introduction

Acetaminophen is a commonly used analgesic and antipyretic drug that induces liver toxicity when used above therapeutic levels. Due to its ease of access, acetaminophen is frequently used in intentional suicide attempts. Acute acetaminophen overdose is the leading cause of acute liver injury (ALI) in the USA and Northern Europe [1]. ALI is a sudden hepatic dysfunction occurring in patients without pre-existing liver disease; it is defined as a coagulopathy with an international normalized ratio (INR) exceeding 1.5. Acute liver failure (ALF) is a more severe condition than ALI, characterized by hepatic encephalopathy with alteration of mental status. ALF can lead to multiorgan failure, and may have a fatal outcome unless appropriate medical management is implemented [2]. Emergency liver transplantation (LT) is the only effective treatment for ALF patients who meet criteria indicative of a poor prognosis.

The models most widely used to predict outcomes for patients with ALF and select those in need of LT are the King’s College criteria (KCC), the Clichy criteria, and the Model for End-Stage Liver Disease (MELD). Among these models, the KCC have the highest specificity [3,4]. However, these criteria are not entirely satisfactory when used to classify patients with late-stage ALF, as they have a limited sensitivity. As a consequence, a certain number of patients not meeting the criteria fail to survive [5]. Parameters allowing earlier prediction of progression toward ALF are thus needed to guide medical practitioners in their decision to perform LT as early as possible, in particular during the ALI stage [6].

One of the pathophysiological features of acetaminophen-induced ALI/ALF is the production of N-acetyl-p-benzoquinone imine (NAPQI)—a toxic metabolite triggering glutathione depletion and hepatocyte necrosis. This phenomenon is associated with the release of inflammatory mediators and intracellular proteins in the blood, which can serve as biomarkers of disease progression [7]. Circulating levels of aspartate (AST) and alanine (ALT) aminotransferases are commonly used as cytolysis biomarkers in acute liver diseases, but the increase in AST and ALT activities is known not to be predictive of ALI/ALF progression [8,9]. In the search for alternative circulating biomarkers, a few promising candidates—such as cytokeratin-18 (CK-18), high-mobility group protein B1 (HMGB1), and Gc-globulin—have been identified in preclinical studies, and are currently being clinically assessed either separately or in combination, along with analysis of composite scores [10,11,12].

In this study, we hypothesized that acetaminophen-induced ALI may be associated with hepatocyte necrosis, followed by leakage of intracellular proteins into the blood, which could be specific indicators of liver damage. Based on this assumption, we used a knowledge-based approach to select potential liver injury candidate biomarkers from available omics information. For this, we implemented a strategy based on bioinformatics tools, and developed a specific selection workflow available via a Galaxy-based instance [13]. Then, we used mass spectrometry (MS)-based discovery proteomics to compare the serum proteomes from acetaminophen-induced ALI patients and healthy donors, and verify the specific leakage of intracellular proteins into the blood. Among the proteins that were significantly increased in the serum of ALI patients, seven were common to the bioinformatics-based selection, including alcohol dehydrogenase 1B (ADH1B), which was detected in high abundance. A total of seven ADH isoenzymes are expressed in the liver [14]. Their specific detection is challenging, as these proteins have a high degree of protein sequence identity, with numerous genetic polymorphisms and overlapping expression patterns. To specifically evaluate ADH1B as a blood biomarker candidate, we used liquid chromatography–selected reaction monitoring (LC–SRM) analysis combined with an isotopically labeled protein standard to specifically detect and reliably quantify ADH1B in serum samples [15]. We then assessed the relevance of ADH1B in tracking acetaminophen-induced ALI/ALF progression in a longitudinal study of serum samples collected from a cohort of 17 patients.

## 2. Results

### 2.1. In Silico Selection of Candidate Biomarkers of Hepatocyte Cell Death

**Figure 1:**
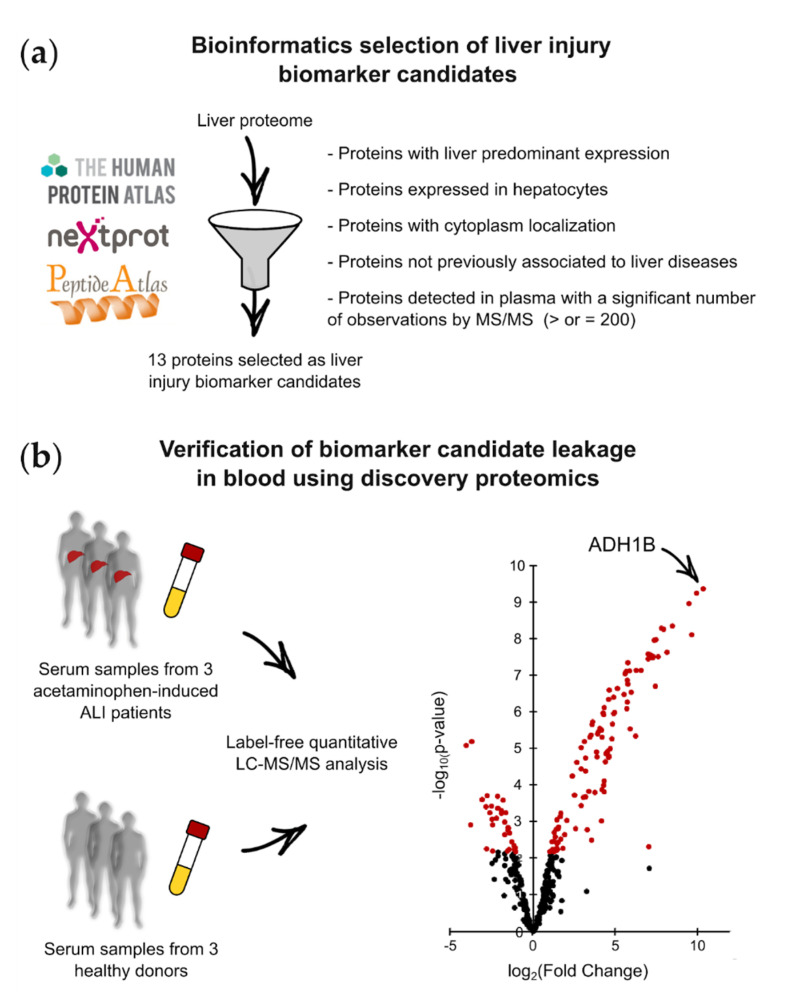
Bioinformatics selection and proteomics discovery of liver injury biomarker candidates: (**a**) Principle and selection criteria to select biomarker candidates for hepatocyte cell death potentially detectable in plasma. (**b**) Label-free quantitative proteomics analysis of serum samples from acetaminophen-induced ALI patients and healthy donors to verify the leakage of biomarker candidates into the blood.

We first closely examined the biological and biochemical characteristics of the cell death biomarkers (also known as cytolysis biomarkers) currently used in medical biology. Most biomarkers are cytoplasmic proteins with a tissue-predominant expression profile and a molecular weight (MW) of less than 85 kDa. Using these features as selection criteria, we mined public databases to extract proteins that could constitute biomarker candidates for liver injury from the liver proteome. Candidate biomarkers for hepatocyte cell death were selected using tools from ProteoRE—a freely accessible Galaxy-based platform (https://www.proteore.org). ProteoRE can be used to implement selection strategies to discover biomarkers of tissue leakage and cancer [13,16]. The tools and workflow implemented for this study are fully documented and available, meaning that others can reuse them (see the “Materials and Methods” section). Candidates were selected following successive steps, as described in Figure 1a. A workflow diagram also shows the ProteoRE tools used and the order in which they were applied in this study (Supplementary Figure S1). First, we established a list of proteins predominantly expressed in the liver, and more specifically in hepatocytes. To produce this list, we combined data from the Human Protein Atlas (HPA), which includes tissue and cellular expression profiles for human proteins based on transcriptomic and immunohistochemical analyses [14]. This double selection was made using the HPA classification of protein-coding genes, with regard to their tissue-restricted expression (based on the three categories “tissue enriched”, “group enriched”, or “tissue enhanced”) [17]. Next, using curated annotations from neXtProt [18], we filtered this list to only retain cytoplasmic proteins with no transmembrane domains and a MW of less than 85 kDa. The “diseases” information extracted from neXtProt was then used to filter out candidates annotated as being associated with diseases other than liver injury. This step helped to exclude non-specific candidates from the final list. Finally, as lack of detectability in biological fluids is a major challenge during biomarker development, we searched for indications that the candidates would be detectable in plasma using MS-based techniques. To do so, MS-based proteomics datasets downloaded from the PeptideAtlas database were mined to check whether these proteins had previously been detected by LC–MS/MS, and to glean information on the total number of peptide observations in plasma [19] (Figure 1a). Candidates not reported as previously being detected by MS in plasma, or those with a low number of peptide observations, were eliminated. The final list of biomarker candidates for liver injury consisted of 13 proteins (Supplementary Table S1).

### 2.2. Verification of Candidate Biomarker Leakage in the Blood of Acetaminophen-Induced ALI Patients

To verify the blood leakage of hepatocyte intracellular proteins during acetaminophen-induced liver injury, we compared the serum proteomes of three ALI patients admitted for acute acetaminophen intoxication and three healthy donors, using label-free quantitative MS-based proteomics. For this unbiased characterization and comparison, serum samples from three acetaminophen-induced ALI patients with ALT serum concentrations >2500 U/L were collected during the early phase of the disease. Serum samples were prepared using an adapted MED–FASP protocol [20], and were analyzed via nano-LC–MS/MS (Figure 1b). Only proteins detected in the three replicates of one condition were considered. This strategy allowed the reliable quantification of 375 unique proteins in the analyzed serum samples (Supplementary Table S2). The differentially abundant proteins in the sera of ALI patients and healthy donors were sorted out by combining a log_2_ (fold change) ≥ 1 or ≤−1, and a limma test *p*-value < 0.0068, allowing us to reach a Benjamini–Hochberg FDR < 1%. Upon applying these parameters, 138 of the 375 unique proteins were identified as being differentially abundant between ALI patients and healthy volunteers. Of these proteins, 105 were significantly more abundant in the sera of acetaminophen-induced ALI patients, among which 7 were shared with the list of biomarker candidates selected using the Galaxy-based bioinformatics workflow (Table 1). As intracellular hepatocyte components, these seven proteins can be considered as tissue leakage biomarkers when detected in the blood. In this study, we further evaluated ADH1B, which displayed the most extensive leakage in the blood of acetaminophen-induced ALI patients (Figure 1b).

### 2.3. Development and Evaluation of ADH1B Targeted Proteomics Assay

**Figure 2:**
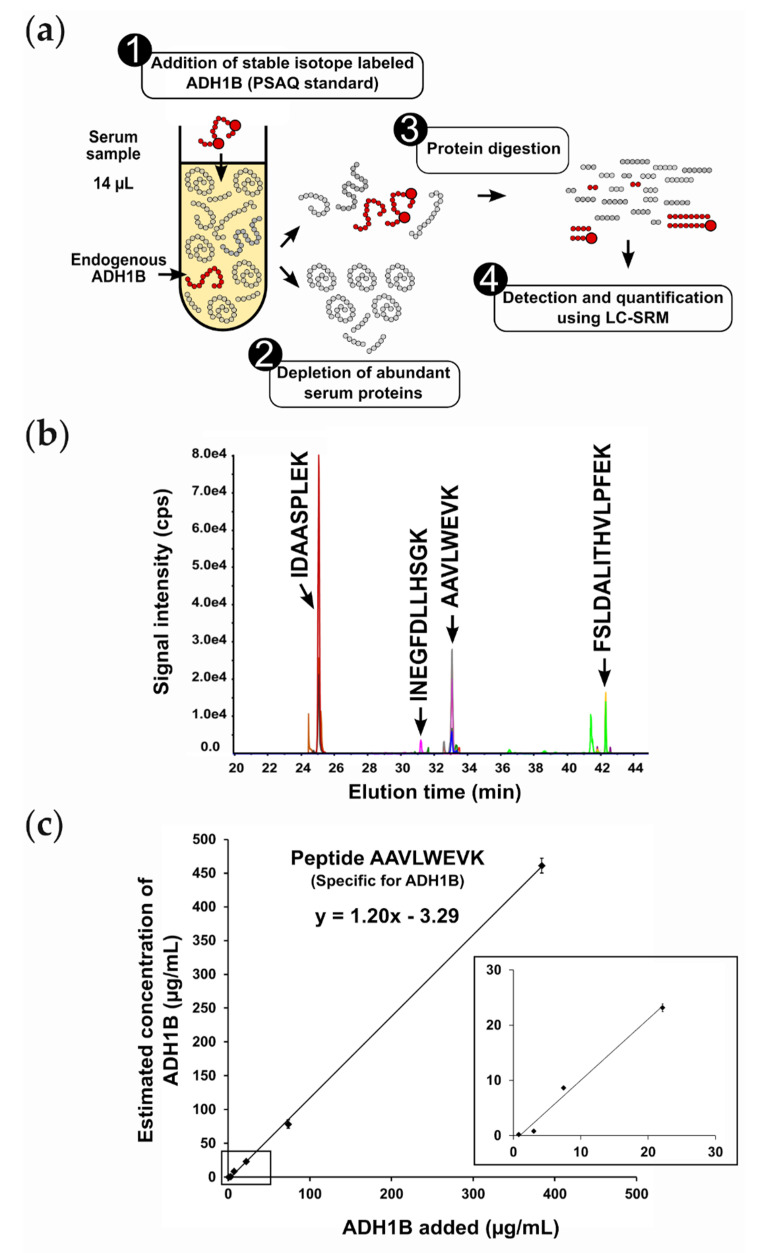
Targeted proteomics assay to quantify ADH1B in serum or plasma samples: (**a**) Different steps of the analytical workflow. (**b**) Extracted ion chromatogram obtained after serum depletion, digestion, and analysis by scheduled LC–SRM. Four peptides generated by ADH1B proteolysis were monitored. Only the specific peptide for ADH1B isoenzymes (peptide AAVLWEVK) was considered for ADH1B quantification. (**c**) ADH1B calibration curve.

To evaluate ADH1B as a new liver injury biomarker candidate, we developed a targeted proteomics assay enabling accurate quantification of ADH1B in serum samples. As ADH1B was barely detected in the three serum samples from healthy donors using MED–FASP preparation and discovery proteomics, we decided to introduce a protein depletion step before proteomics analysis in order to improve detection sensitivity in these samples. To develop the targeted proteomics assay, serum samples were spiked with recombinant ADH1B, depleted, and digested before LC–SRM analysis to select surrogate peptides with high detectability in the serum matrix, in order to determine the best transitions for each surrogate peptide and to schedule acquisition (Supplementary Table S3). For each peptide detected, sequence uniqueness was verified via BLAST search against the UniProt database. Among the four peptides detected, only one was found to be truly specific to ADH1B isoenzymes (peptide AAVLWEVK) (Supplementary Figure S2). We also took genetic variation into account, and searched for non-synonymous single-nucleotide polymorphisms [21]. Thankfully, the peptide AAVLWEVK was shared between the three variants of ADH1B associated with genetic polymorphisms—namely, ADH1B*1, ADH1B*2, and ADH1B*3—and, consequently, can be used for patients from any population [22]. To quantify ADH1B, we synthesized and used a PSAQ standard, which is a full-length isotopically labeled version of the target analyte. The choice of a PSAQ standard was essential to accurately quantify ADH1B, as sample prefractionation and proteolysis processes can lead to analyte losses [15,23]. The final LC–SRM assay involved spiking samples with ADH1B PSAQ standard, the depletion of abundant proteins, protein digestion, and LC–SRM analysis (Figure 2a,b).

As recommended by the bioanalytical and proteomics communities [24,25,26], we assessed the analytical performance characteristics of ADH1B via targeted proteomics assay (Table 2, Supplementary Figure S3). Firstly, the assay was assessed for matrix effects and interferences. The impact of the blood sampling procedure (plasma or serum specimens) and interfering biological substances (hemoglobin, triglycerides, and bilirubin) was evaluated in parallel experiments, where a controlled mix of recombinant ADH1B and labeled ADH1B were spiked into the different biological samples. The concentrations of ADH1B measured in the individual samples (*n* = 2 full technical replicates per sample) differed by no more than 14% compared to the initial mix, except for hemolytic samples, in which pre-analytical interference was more important (24%).

Linearity, accuracy, LLOD, LLOQ, and technical precision were determined from a calibration curve using six non-zero standards between 1 and 384 µg/mL (*n* = 3 or 4 full technical replicates per calibration point) (Figure 2c, Supplementary Figure S3). The ADH1B signature peptide AAVLWEVK provided excellent analytical performance, with an accuracy (trueness) of 120% and perfect linearity (R^2^ = 1) over the whole measurement range. LLOD and LLOQ were determined from the calibration curve using the calibration plot method [27], and were estimated at 2 µg/mL and 6 µg/mL, respectively. Technical precision at the LLOQ was 8%.

Assay repeatability and reproducibility were assessed using a “disease” pool (*n* = 20 pooled serum samples from ALI patients) and a “healthy” pool (*n* = 20 pooled serum samples from healthy donors), which were spiked with a defined quantity of labeled ADH1B. Then, the endogenous concentrations of ADH1B were measured in five technical replicates of both pools analyzed on the same day, and five technical replicates analyzed on five successive days. Based on the “disease” pool results, the intraday and interday assay variability was 3.2% and 4.2% respectively (Table 2, Supplementary Figure S3). ADH1B was undetectable in the “healthy” pool.

Sample handling stability was also assessed using the “disease” pool. For these assays, the pooled sample was divided into several aliquots that were spiked with a defined quantity of labeled ADH1B before biochemical processing. The peptide digests obtained were analyzed under a range of conditions (*n* = 2 full technical replicates per condition): (1) after storing samples at room temperature (21 °C) for 4 h, (2) after storage at 4 °C for 24 h, (3) after two freeze–thaw cycles, and (4) after frozen storage for 30 days at −20 °C. Endogenous concentrations of ADH1B were estimated in the different replicates, and the differences (%) between each condition and immediate analysis were determined. In these stressed storage conditions, these experiments indicated that ADH1B quantification varied by no more than 2.2% (Table 2, Supplementary Figure S3). Analyte stability was estimated at 19.8% by measuring ADH1B in two clinical samples before and after storage at −80 °C for 3 months.

Finally, the stability of the signature peptide AAVLWEVK after sample digestion was specifically assessed using a double-labeled peptide as standard, with a different mass from the one generated by the labeled ADH1B standard. The stability was assessed under four different storage conditions (*n* = 3 full technical replicates per condition): (1) after sample storage at room temperature (21 °C) for 4 h, (2) after storage at 4 °C for 24 h (autosampler stability), (3) after two freeze–thaw cycles, and (4) after frozen storage at −80 °C for >30 days. The results of these experiments demonstrate that the AAVLWEVK peptide was stable (CV < 10%) under these conditions (Table 2, Supplementary Figure S3).

### 2.4. Blood Leakage Pattern of ADH1B in Acetaminophen-Induced ALI

**Figure 3:**
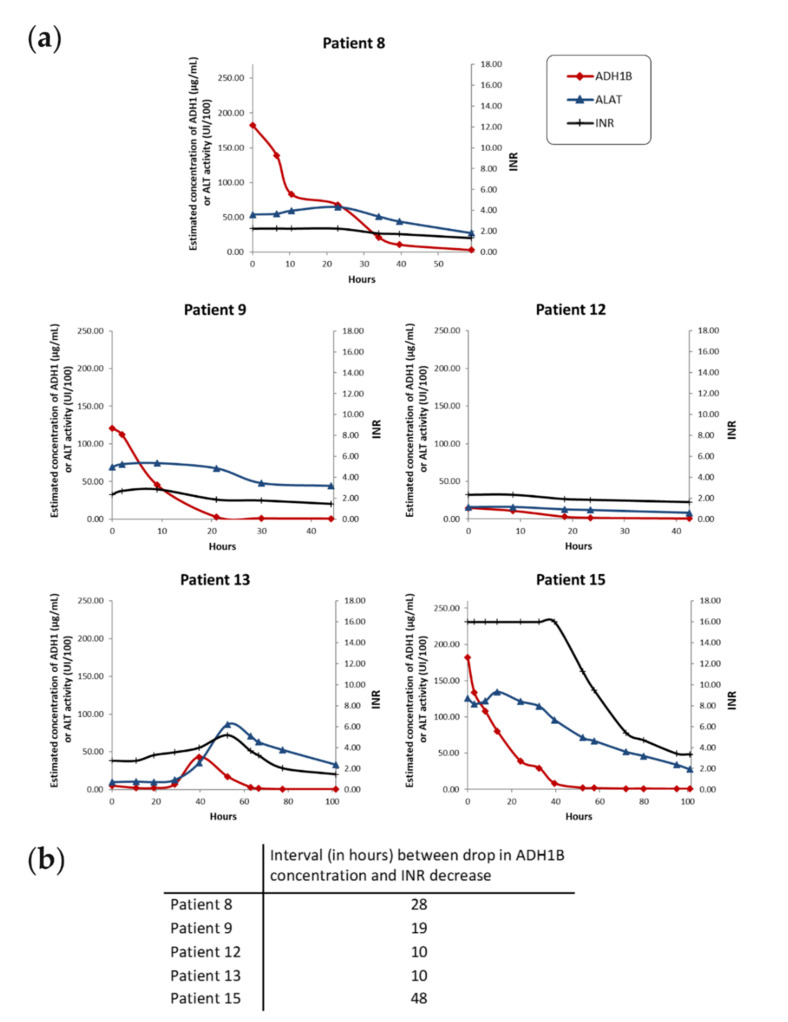
Time-dependent changes in liver injury biomarkers in five patients with acetaminophen-induced ALI/ALF. In these five patients who recovered spontaneously, ADH1B serum concentration started to decrease before the INR decreased. (**a**) Biological profiles for ALI/ALF patients who recovered spontaneously. ADH1B serum concentration was determined via quantitative LC–SRM. The INR indicates coagulation defects. Serum ALT activity is also indicated for comparison. Data points were connected using a smoothed line. (**b**) Interval between the earliest drop in ADH1B serum concentration after peaking and the decrease in the INR.

Once the analytical pipeline had been optimized, we characterized the ADH1B serum profile over the course of acetaminophen-induced ALI/ALF. For these assays, we recruited a cohort of 17 patients hospitalized for acetaminophen-induced ALI. Diagnosis of ALI was based on the absence of pre-existing liver disease, evidence of acetaminophen ingestion, absence of encephalopathy, an INR of 2.0 or greater, and elevated ALT. All patients were treated with N-acetylcysteine (NAC) until they recovered spontaneously or received an LT. Fourteen patients recovered spontaneously, five progressed to ALF with evidence of hepatic encephalopathy, and three underwent LT (Supplementary Table S4). ADH1B levels were quantified in serum samples collected both at the time of patient admission and as their ALI progressed, when blood sampling was required for medical management of the patients. The time-course for serum ADH1B concentrations was compared to the profile for standard biological indicators of liver dysfunction, including INR and ALT activity (Figure 3a, Supplementary Table S4, Supplementary Figure S4). To determine physiological levels of ADH1B, serum samples from seven healthy donors were also analyzed.

ADH1B was undetectable in sera from healthy donors (Supplementary Table S4). In contrast, significant levels of all of the biomarkers monitored were detected in most samples from patients with acetaminophen-induced ALI (Figure 3a, Supplementary Table S4, Supplementary Figure S4). On admission, ADH1B concentrations in patient samples ranged from 2 to 204 µg/mL. Levels of ADH1B increased as the disease progressed, along with INR, bilirubin, and aminotransferases. Unlike ALT activity, serum ADH1B dropped to undetectable levels during the recovery period, indicating that ADH1B is a specific marker of liver cytolysis. During the acute phase of ALI, the serum concentrations of ADH1B were of the same order of magnitude as concentrations of major serum proteins such as transferrin and IgA (≈100 µg/mL) [28]. Circulating levels of ADH1B varied extensively, and were synchronized with INR scores in nine patients (patients 1–7, 10, and 17) who spontaneously recovered (Supplementary Figure S4). In the other five patients (patients 8, 9, 12, 13, 15) who spontaneously recovered, analysis of closely spaced timepoints revealed that the ADH1B serum concentration started to decrease long before the INR decreased (between 10 and 48 h), in reflection of the onset of liver recovery (Figure 3b). This result shows that, at least in some patients, ADH1B is an earlier indicator of ALI recovery than the standard biological parameters. Along this line, four patients (patients 1, 6, 15, and 17) had KCC with MELD scores >40, which are considered to reflect a poor prognosis unless LT is performed, but nevertheless survived without transplantation. Interestingly, in these patients, the decrease in ADH1B serum levels associated with INR improvement correspondingly indicated favorable outcomes.

**Figure 4:**
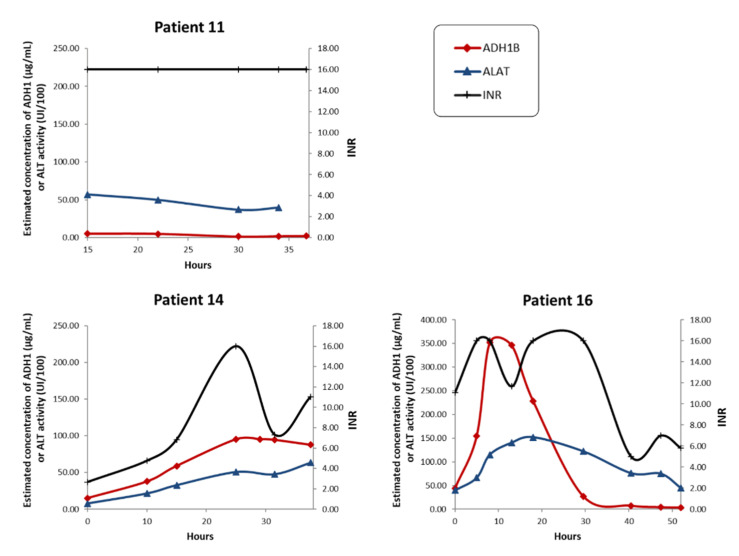
Time-dependent changes in liver injury biomarkers in patients with acetaminophen-induced ALI/ALF who received a liver transplant. Biological profiles of ALI/ALF patients before liver transplantation. ADH1B serum concentration was determined via quantitative LC–SRM. The INR is reported as an indicator of coagulation defects. Serum ALT activity is also indicated for comparison. Data points were connected using a smoothed line.

In two patients (patients 11 and 16) who developed ALF and underwent LT, the dramatic scores for the standard biological parameters (INR, bilirubin, and creatinine) were indicative of severe liver failure and associated with low concentrations of ADH1B from the time of hospital admission (patient 11), or after the cytolytic episode (patient 16) (Figure 4). Since ADH1B specifically reflects hepatocyte cytolysis, this profile suggests a massive loss of hepatocytes, resulting in reduced biomarker leakage at the end stages (depleted pool of ADH1B) compared to the early stages of ALF.

In summary, ADH1B is a potential biomarker of liver injury, specifically leaked into the blood flow following hepatocyte cytolysis. ADH1B can be used to monitor the evolution of acetaminophen-induced ALI. In patients who recover spontaneously, an ADH1B assay can detect the onset of recovery when the INR starts to drop, or even earlier.

### 2.5. Blood Leakage Pattern of ADH1B in Non-Acetaminophen-Induced ALI

**Figure 5:**
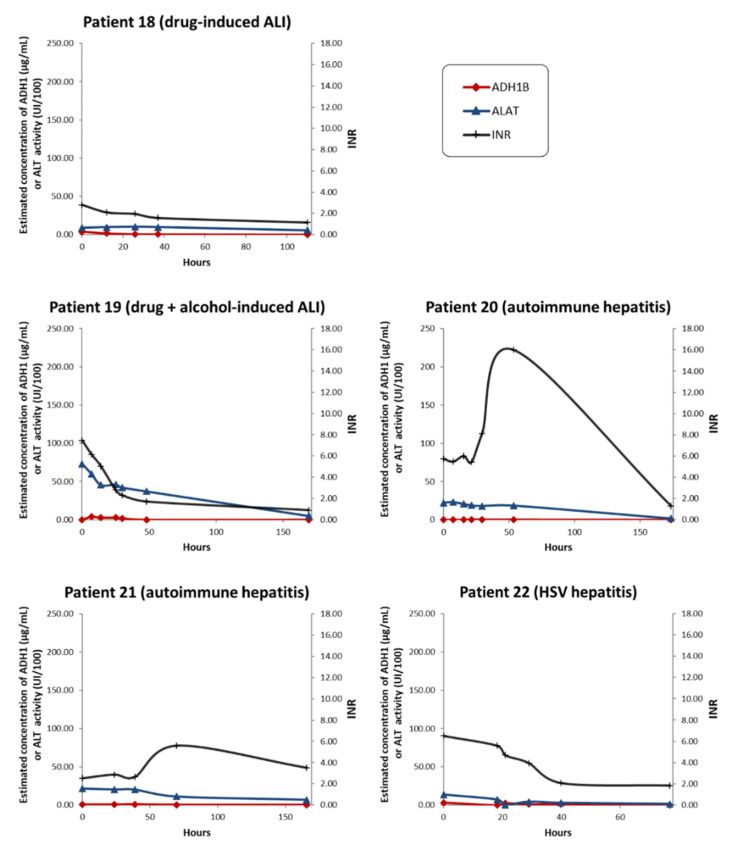
Time-dependent changes in liver injury biomarkers in patients with non-acetaminophen-induced ALI/ALF. ADH1B serum concentration was determined via quantitative LC–SRM. ALI/ALF etiology is indicated on each graph. The INR is reported as an indicator of coagulation defects. Serum ALT activity is also indicated for comparison. Data points were connected using a smoothed line.

To verify the specificity of ADH1B as a marker for acetaminophen-induced ALI, we investigated the time-course of ADH1B serum levels in patients with non-acetaminophen-induced ALI (*n* = 5). Several types of ALI were considered, including drug-induced ALI, autoimmune hepatitis, and herpes simplex virus (HSV)-induced hepatitis (Figure 5, Supplementary Table S4). ADH1B could not be detected in any of the serum samples collected from the two patients with autoimmune hepatitis before LT, although serum transaminases were elevated and the INR was >2.5 (Figure 5, Supplementary Table S4). With drug-induced ALI and HSV-induced hepatitis, circulating ADH1B was detected at very low levels (below LLOQ) at the earliest timepoints of the disease. ADH1B could be quantified at 12.6 µg/mL in a single serum sample from patient 19 (drug-induced + alcohol-related ALI) at hospital admission. Even though more extensive sampling would be necessary to draw firm conclusions, massive ADH1B leakage into the blood appears to be specifically correlated with acetaminophen-induced ALI/ALF.

## 3. Discussion

Multiomics integration, trans-omics, and artificial intelligence methods hold considerable potential in medicine to explore massive omics data and deliver new biomarker candidates and drug targets [29,30,31]. Along this line, recent studies have exploited proteomic maps to select tissue-specific proteins as biomarker candidates. In 2012, Prassas et al. [32] combined data deposited in transcriptomics and proteomics databases to identify proteins with tissue-specific expression that could be relevant biomarkers secreted in cancers. In the field of hepatology, Qin et al. [33] recently selected a panel of 66 human proteins based solely on their liver expression profiles. The levels of 23 of these proteins were demonstrated to increase in 14 serum samples from patients with acetaminophen overdose, but the potential clinical value of these proteins was not further explored. In this study, we applied a similar initial hypothesis, and selected 13 proteins from omics databases as potential highly specific candidate biomarkers to monitor acetaminophen-induced ALI progression. Label-free quantitative MS-based proteomics was used to refine the selection and identify intracellular proteins specifically released into the blood as a result of hepatocyte necrosis and liver damage. Extensive leakage of ADH1B into the circulation was evidenced, motivating the further study of ADH1B serum concentrations in acetaminophen-induced ALI patients using targeted quantitative proteomics. 

In accordance with our hypothesis and the label-free proteomics results, longitudinal monitoring of patients with acetaminophen-induced liver injury confirmed extensive leakage of ADH1B into the circulation during the acute phase of the disease. Patients who spontaneously recovered had a time-course of ADH1B serum concentrations that closely mirrored their clinical status and, unlike ALT enzymatic activity, ADH1B serum levels rapidly returned to baseline during liver regeneration. Furthermore, likely because it has a very short half-life in blood, the ADH1B concentration rapidly and markedly decreased following the end of hepatocyte cytolysis. Thanks to close biological monitoring, we also found that the decrease in circulating ADH1B levels preceded recovery of liver function in 5 out of 14 cases of spontaneous recovery. The interval between the drop in ADH1B levels and the INR decrease was between 10 and 48 h. From these data, ADH1B appears to be a biomarker of hepatocellular injury that can be used to monitor acetaminophen-induced ALI/ALF progression. In particular, the rapid drop in ADH1B serum concentrations, followed by a subsequent decrease in the INR, appears to be an early indicator of spontaneous ALI resolution.

Recently, Dear et al. [34] investigated the potential of new mechanistic biomarkers—including mir-122, HMGB1, CK-18, and glutamate dehydrogenase (GLDH)—to stratify patients at risk of liver injury into two large cohorts of patients with paracetamol overdose and various clinical presentations. Among these biomarkers, HMGB1 (which reflects hepatocyte necrosis and immune cell activation) was able to predict an increase in INR of over 1.5 at hospital admission. From these results, HMGB1 is expected to provide useful information to identify patients at risk of ALF and death. Another recent study by Nuzzo et al. [35] also revealed that the plasma levels of procalcitonin on admission may be an early independent predictor of liver injury. In this context, combinations of mechanistic biomarkers such as ADH1B, HMGB1, and functional biomarkers such as INR may improve the prediction of acetaminophen-induced ALI evolution, as well as patient stratification and management. Along this line, ADH4—which was also selected using our methodology (Table 1)—could also be considered as a potential liver injury biomarker.

In conclusion, in this study we established a link between large-scale omics data (proteomics, transcriptomics), bioinformatics, and MS-based proteomics to deliver a new biomarker candidate for hepatology and clinical toxicology. Specifically, a dedicated bioinformatics workflow was developed to extract mechanistic biomarker candidates from public “omics” databases. Then, discovery and targeted proteomics analyses were sequentially used to identify and quantify ADH1B in serum samples from patients with acetaminophen-induced ALI. This isoenzyme, rarely accessible via immunoassays, was revealed as being useful to monitor the progression of acetaminophen-induced ALI/ALF. Our results also emphasize the potential of combining ADH1B and INR measurements to better assess the progression of acetaminophen-induced ALI/ALF.

## 4. Materials and Methods

### 4.1. Bioinformatics Selection of Candidate Biomarkers

The tools implemented and used in this study are part of the freely accessible ProteoRE platform (https://www.proteore.org)—a Galaxy-based platform dedicated to the functional analysis and exploration of biomedical-research-related proteomics data. The ProteoRE tools used in this study were the following: “Data manipulation and visualization” section: “ID Converter (Human, Mouse, Rat)”, “Filter by keywords and/or numerical value”, and “Venn diagram (JVenn)”; “Get features/annotation” section: “Build tissue-specific expression dataset (Human Protein Atlas)(no input required)”, “Add expression data (RNAseq or Immuno-assays) (Human Protein Atlas)”, “Get expression profiles by (normal or tumor) tissue/cell type (Human Protein Atlas)”, “Add protein features (neXtProt)”, and “Get MS/MS observations in tissue/fluid (Peptide Atlas)”. For ease of use, detailed user documentation is provided at the bottom of the central panel of the Galaxy interface upon tool selection. This documentation describes what the tool does, the input it requires, the parameters to be tuned, and the output it produces. Data sources are listed along with their release date, when applicable. The computational outputs—history, workflow, and tools—used and produced in this study have been published and shared via ProteoRE to allow for review and to make them available for reuse. These resources are available in the shared section (menu “Shared data” from the navigation bar on the main panel) on the ProteoRE website (registered users only). The history and workflow for this study can also be examined and imported by accessing the following links: http://www.proteore.org/u/yvdb/h/liverinjurybiomarkersselectionpailleuxetal (name: “LiverInjury_Biomarkers_Selection_Pailleux_et_al”) and http://www.proteore.org/u/yvdb/w/workflow-constructed-from-history-pailleuxetalliverinjurybiomarkersselection (name: “Workflow ‘LiverInjury_Biomarkers_Selection’ Pailleux_et_al”. The workflow can be reused by clicking the “import workflow” button (upper-right corner of the “About this Workflow” panel). The public resources exploited (content and release information) were the following: human tissue expression profiles for transcripts and proteins based on RNA sequencing analysis and immunohistochemistry, respectively, downloaded from the Human Protein Atlas (HPA) (https://www.proteinatlas.org/about/download; version 20.1) and Ensembl (version 92.38). Protein annotations were retrieved from neXtProt (release May 2019) using the REST application programming interface (https://api.nextprot.org/). Proteomics builds (i.e., collections of peptides identified in samples from a particular subproteome generated by PeptideAtlas (https://db.systemsbiology.net/sbeams/cgi/PeptideAtlas/buildInfo)) were retrieved via the PeptideAtlas query interface. Information on the human plasma proteome build used in this study (release date April 2017) can be found here: https://db.systemsbiology.net/sbeams/cgi/PeptideAtlas/buildDetails?atlas_build_id=465.

### 4.2. Human Blood Samples

Patients with ALI/ALF were recruited at the Intensive Care Unit (ICU) of the Hepatobiliary Center at Paul Brousse Hospital (France, Villejuif). Blood samples were collected from 22 patients admitted for acetaminophen-induced ALI/ALF (*n* = 17) and non-acetaminophen-induced ALI/ALF (*n* = 5) (Supplementary Table S4). Blood samples were analyzed immediately at the clinical chemistry laboratory to determine biological parameters of hepatic insufficiency, including alanine and aspartate aminotransferase (activated ALT and AST photometric assays, Abbott, Rungis, France), INR, and prothrombin time (STA-Neoplastine CI PLUS test, Ref. 00667, Diagnostica Stago, Asnières sur Seine, France). All patients tested negative for hepatitis viruses (HAV, HBV, HCV). Clinical data collected included age, gender, sampling time, and outcome on day 21 post-admission (Supplementary Table S4). Blood samples were collected at the time of admission to the ICU, and several times per day for several consecutive days until clinical improvement, full recovery, or LT. For proteomics analyses, blood samples were collected in non-treated tubes (BD Biosciences, Le Pont de Claix, France), and serum was isolated by centrifugation at 1000 g for 15 min in the 30 min following collection. Aliquots of serum supernatants were immediately frozen at −80 °C before shipping to the proteomics laboratory in dry ice. After receipt in the proteomics laboratory, samples were thawed and aliquoted (50 µL) before storage at −80 °C. The French Blood Service (EFS) provided anonymous serum samples from healthy donors (*n* = 20) with no history of acetaminophen-induced hepatotoxicity.

### 4.3. ADH1B Recombinant Protein and Quantification Standard

Unlabeled recombinant ADH1B protein was purchased from Abcam, Cambridge, UK (reference ab116934). The protein standard for absolute quantification (PSAQ) for ADH1B was synthesized using a cell-free protein expression system in the presence of [^13^C_6_, ^15^N_2_] L-lysine and [^13^C_6_, ^15^N_4_] L-arginine (Euriso-top, Saint-Aubin, France), as previously described [36,37]. The ADH1B PSAQ standard was checked for purity (>95%, SDS-PAGE) and isotope incorporation via LC–SRM (>99%), before quantification by amino acid analysis [38]. Purified ADH1B PSAQ standard was formulated in phosphate-buffered saline, and was aliquoted in single-use low-binding microtubes (Eppendorf, Montesson, France) to avoid freeze–thaw cycles. Aliquots were stored at −80 °C.

### 4.4. Preparation of Serum Samples for Discovery Proteomics Analysis

Serum samples were digested using multiple-enzyme digestion–filter-aided sample preparation (MED–FASP) [20], with slight variations to the protocol as previously described [39]. Briefly, each serum sample (3 µL) was loaded on a 10 kDa cutoff ultrafiltration device (Amicon). Proteins were denatured and reduced on the device in 4 M urea, 25 mM NH_4_HCO_3_, and 20 mM TCEP. The sample was washed with 4 M urea and 25 mM NH_4_HCO_3_ before performing alkylation in 4 M urea, 25 mM NH_4_HCO_3_, and 55 mM iodoacetamide. After two additional washing steps, the sample volume was reduced to 50 μL, and proteins were digested for 2 h at 37 °C using trypsin/LysC mix (Promega) at a protein/enzyme ratio of 1:20 (*w*/*w*). The urea concentration was reduced below 1 M, and digestion was allowed to proceed for 5 h at 37 °C. Proteolytic peptides were recovered by adding 100 µL of 25 mM NH_4_HCO_3_ to the filter and centrifuging for 15 min at 12,000× *g* and 4 °C. The peptide digest was purified on C18 Macro SpinColumns (Harvard Apparatus, Les Ulis, France), and 5 µg aliquots of each sample were dried by vacuum centrifugation.

### 4.5. MS-Based Discovery Proteomic Analyses

Dried peptide digests were solubilized in 20 µL of 5% acetonitrile, 0.1% trifluoroacetic acid, containing 0.5 UI/µL of HRM-iRT. Then, 2 μL (equivalent to ≈500 ng protein) of this solution was analyzed via nano-LC–MS/MS (Ultimate 3000 nano RSLC and Q Exactive HF equipped with Nanospray Flex Ion Source, Thermo Fisher Scientific, Les Ulis, France). Peptides were sampled on an Acclaim™ PepMap™ 100 C18 300 µm × 5 mm precolumn (Thermo Fisher Scientific) and separated on a ReproSil-Pur 120 C18-AQ, 1.9 μm, 75 µm × 25 cm column (Dr. Maisch GmbH). The nano-LC method consisted of a 60 min multilinear gradient ranging from 4% to 50% solvent B (80% acetonitrile, 20% water, 0.1% formic acid) at a flow rate of 300 nL/min, with the column oven at 35 °C. The column was then washed with 90% solvent B for 15 min and, finally, re-equilibrated with 4% solvent A (2% acetonitrile, 98% water, 0.1% formic acid) for 15 min. MS and MS/MS data were acquired using Xcalibur (Thermo Fisher Scientific). A data-dependent top-20 MS acquisition method was launched with the Q Exactive HF in positive mode. The nanospray voltage was set to 2 kV, the ion transfer capillary temperature to 270 °C, and the S-Lens RF amplitude to 55%. Survey full-scan MS spectra (m/z = 400–1600) were acquired in the Orbitrap at a resolution of 60,000 after the accumulation of 10^6^ ions (maximum filling time: 200 ms). The 20 most intense ions (excluding z = 1 and unassigned charge states) from the survey scan were fragmented using HCD with a normalized collision energy of 30, a resolution of 15,000, and an AGC target of 10^5^ ions (max injection time: 50 ms). Dynamic exclusion was set to 30 s, the intensity threshold to trigger MS/MS to 2 × 10^4^, and the default charge state to 2.

### 4.6. Discovery Proteomics Data Processing

Peptides and proteins were identified using Mascot (version 2.7.0.1) via concomitant searches against the UniProt database (*Homo sapiens* taxonomy, downloaded in May 2021) and a homemade database of frequently observed non-human contaminants. Trypsin/P was chosen as the enzyme, and two missed cleavages were allowed. Precursor and fragment mass error tolerances were set at 10 and 20 ppm, respectively. Peptide modifications allowed during the search were carbamidomethyl (C, fixed), acetyl (protein N-term, variable), and oxidation (M, variable). The Proline software [40] was then used to filter the results: conservation of rank 1 peptides; peptide length ≥ 6 amino acids; peptide score ≥ 25; ability to reach a false discovery rate of peptide-spectrum match identifications < 1%, as calculated based on peptide-spectrum match scores by employing the reverse database strategy. Proline was then used to combine all validated PSMs into a single final mapping list of proteins. Only proteins with at least one specific peptide were kept. MS1 label-free quantification of the identified protein groups based on razor and specific peptides was finally performed with Proline.

Statistical analyses were performed using ProStaR [41]. Proteins identified in the reverse and contaminant databases, proteins identified by MS/MS in less than two replicates of one condition, and proteins for which fewer than 3 abundance values were available in a single condition were removed from the list. After log_2_ transformation, abundance values were normalized by VSN (variance-stabilizing normalization) before imputing missing values (SLSA algorithm for partially observed values in the condition, and detQuantile algorithm for totally absent values in the condition); statistical testing was performed using a limma-moderated t-test. Differentially recovered proteins were sorted out using a log_2_ (fold change) cutoff of 1 and a *p*-value threshold (on the remaining proteins) that guarantees a Benjamini–Hochberg FDR <1%.

### 4.7. Preparation of Serum Samples for Targeted Proteomics Analysis

Serum samples (14 µL) were spiked with defined amounts of ADH1B PSAQ standard (3 µg/mL), and then incubated for 1 h at 4 °C with gentle mixing. Spiked samples were depleted of the six most abundant proteins (albumin, transferrin, IgG, IgA, haptoglobin, and antitrypsin) using a human Multiple Affinity Removal System (MARS) spin cartridge (Agilent Technologies), in accordance with the manufacturer’s instructions. Depleted samples were concentrated to 50 µL, and the buffer was exchanged for 4 M urea and 50 mM NH_4_HCO_3_ using a 3000 Da cutoff ultrafiltration device (Merck Millipore, Guyancourt, France). The resulting concentrates were submitted to in-solution digestion at 37 °C using an EndolysC/trypsin mix (Promega, Charbonnières-les-Bains, France) at an enzyme/protein ratio of 1:30 (*w*/*w*). After incubation for 3 h, samples were diluted (4×), and digestion was allowed to proceed overnight at 37 °C. Digestion was stopped by adding 0.1% formic acid. Samples were purified on C18 Macro SpinColumns (Harvard Apparatus) and dried by vacuum centrifugation. Digests were resolubilized in 15 µL of 2% acetonitrile, 0.1% formic acid. A sample volume of 6 µL was injected into the LC system.

### 4.8. LC–SRM Analysis

Targeted proteomics analyses were performed in SRM mode on a QTRAP 6500 mass spectrometer (AB Sciex, Darmstadt, Germany) (400–1000 *m*/*z* range) equipped with a TurboV source and controlled by Analyst software (version 1.6.1, AB Sciex). The instrument was coupled to an Ultimate 3000 LC chromatography system (Thermo Fisher Scientific). Chromatography was performed using a two-solvent system (solvent A (2% acetonitrile, 0.1% formic acid); solvent B (80% acetonitrile, 0.1% formic acid)). Peptide digests were concentrated on a 1 × 15 mm C18 PepMap precolumn (Thermo Fisher Scientific) before separation on a Kinetex XB-C18 column (2.1 × 100 mm, 1.7 µm, 100 Å; Phenomenex, Le Pecq, France). Separation was performed over 40 min by applying a gradient from 3% to 35% B in 30 min, and from 35% to 90% B in 10 min, at a flow rate of 50 µL/min. The tryptic peptides monitored and the parameters for LC–SRM acquisitions are presented in Supplementary Table S3. A calibration sample was used before LC–SRM analysis of samples from ALI patients to verify quantification performance. Clinical samples were analyzed in batches, in an unblinded fashion, with each batch corresponding to the serum samples obtained from a single patient. Quality controls to check instrument performance and to adjust retention time windows were implemented before each batch of samples. Sample vials and injection plates were maintained at 4 °C in the autosampler, and storage in the autosampler never exceeded 24 h (maximum 13 samples for patient 15). Absence of carryover was checked using a blank injection after each serum sample.

### 4.9. Analysis of LC-SRM Data

LC–SRM data were analyzed using Skyline software (version 19.1.0.193) [42]. The signature peptide AAVLWEVK was used to quantify ADH1B. Other peptides generated by ADH1B proteolysis were also monitored, but they were not taken into account for ADH1B quantification in clinical samples, as they are shared between several protein isoforms (Supplementary Figure S2). Each version (unlabeled/labeled) of the peptide AAVLWEVK was monitored using three transitions (Supplementary Table S3). All transitions were inspected (signal-to-noise ratio, asymmetric peak due to interference, co-elution…) and integration boundaries were manually adjusted before peak area measurements. Unlabeled/labeled peak area ratio was calculated for the most intense transition (quantifier transition), and this ratio was used to calculate ADH1B concentration.

## 5. Patent

The results and biomarker candidates reported in this article are protected by patent WO2017/121974 Process for in vitro diagnosis of hepatic disorders.

## Figures and Tables

**Table 1:** Biomarker candidates for hepatocyte cell death selected using the bioinformatics workflow that are significantly increased in serum samples from acetaminophen-induced ALI patients (*n* = 3).

UniProt ID	Protein Name	Log_2_ (Fold Change)	*p*-Value
P00325	Alcohol dehydrogenase 1B	10.3	4.5 × 10^−10^
P08319	Alcohol dehydrogenase 4	9.9	5.9 × 10^−10^
P00352	Retinal dehydrogenase 1	9.5	1.1 × 10^−09^
P04406	Glyceraldehyde-3-phosphate dehydrogenase	7.3	3.2 × 10^−08^
P24298	Alanine aminotransferase 1	5.7	8.5 × 10^−07^
P78417	Glutathione S-transferase omega 1	5.7	1.4 × 10^−07^
Q3LXA3	Triokinase and FMN cyclase	4.6	1.8 × 10^−05^

**Table 2:** Analytical performance of ADH1B targeted proteomics assay.

Performance Parameter	Result
Matrix effects and interferences (bias)	
Pool of plasma from five healthy donors	−10.0%
Pool of serum from six healthy donors	−14.0%
Hemolytic serum (hemoglobin 500 mg/dL)	−24.0%
Lipemic serum (triglycerides 200 mg/dL)	−14.0%
Lipemic serum (triglycerides 500 mg/dL)	0.0%
Serum with medium bilirubin (82 µmol/L)	−14.0%
Serum with high bilirubin (326 µmol/L)	−10.0%
Calibration curve	
Range of tested concentrations	1 to 384 µg/mL
Accuracy (trueness)	120.0%
Linearity (R^2^)	1.00
LLOD	2 µg/mL
LLOQ	6 µg/mL
Technical precision at LLOQ (CV, *n* = 3)	8.0%
Repeatability and reproducibility	
Intraday (CV, *n* = 5)	3.2%
Interday (CV, *n* = 5)	4.2%
Sample handling stability (bias)	
Sample storage for 4 h at room temperature	−2.2%
Sample storage 24 h at 4 °C	1.8%
Two freeze–thaw cycles	1.0%
Frozen sample storage (−20 °C) for 30 days	1.4%
Analyte stability (bias)	
3 months at −80 °C (*n* = 2 samples from ALI patients)	19.8%
Peptide stability (bias)	
Sample storage for 4 h at room temperature	3.7%
Sample storage 24 h at 4 °C	3.7%
Two freeze–thaw cycles	0.0%
Frozen sample storage (−80 °C) for >30 days	7.4%

## Data Availability

The LC–MS/MS data have been submitted to the ProteomeXchange Consortium via the PRIDE [43] partner repository under dataset identifier PXD028082 (Reviewer account details: Username: reviewer_pxd028082@ebi.ac.uk; Password: IfSnG15F). All LC–SRM raw data (.wiff files), Skyline files (.sky files), and calculations (.xlsx files) have been deposited in the PeptideAtlas SRM Experiment Library (PASSEL) under dataset identifier PASS01148 (password TX8572wv).

## Supplementary Materials

The following are available online at https://www.mdpi.com/article/10.3390/ijms222011071/s1.
