# Supplementary material for: Mass Spectrometry-Based Proteomics Reveal Alcohol Dehydrogenase 1B as a Blood Biomarker Candidate to Monitor Acetaminophen-Induced Liver Injury
Source: Int J Mol Sci. 2021 Oct 14;22(20):11071. doi: 10.3390/ijms222011071 (PMC8540689; doi:10.3390/ijms222011071)
Supplement: Supplementary file 1 [file ijms-22-11071-s001.zip › ijms-1377388-supplementary.pdf]

SUPPLEMENTARY FIGURES

Mass spectrometry-based proteomics reveal alcohol dehydrogenase 1B as a blood biomarker candidate to monitor acetaminophen-induced liver injury

Floriane Pailleux [1,2], Pauline Maes [1,2], Michel Jaquinod [1,2], Justine Barthelon [1,2,3], Marion Darnaud [4,5], Claire Lacoste [4,5], Yves Vandenbrouck [1,2], Benoît Gilquin [1,2,6], Mathilde Louwagie [1,2], Anne-Marie Hesse [1,2], Alexandra Kraut [1,2], Jérôme Garin [1,2], Vincent Leroy [3,7], Jean-Pierre Zarski [3,7], Christophe Bruley [1,2], Yohann Couté [1,2], Didier Samuel [4,5], Philippe Ichai [4,5], Jamila Faivre [4,5,8]*, Virginie Brun [1,2,6]*

[1] Univ. Grenoble Alpes, CEA, Inserm, IRIG, BioSanté U1292, Grenoble, France
[2] Proteomics French Infrastructure, FR2048 CNRS/CEA, Grenoble, France
[3] Clinique Universitaire d'Hépato-gastroentérologie, Centre Hospitalier Universitaire Grenoble, Grenoble, France
[4] Inserm, U1193, Paul-Brousse University Hospital, Hepatobiliary Centre, Villejuif, France
[5] Univ. Paris-Sud, Université Paris-Saclay, Faculté de Médecine Le Kremlin-Bicêtre, France
[6] Univ. Grenoble Alpes, CEA, LETI, Clinatec, Grenoble, France
[7] Univ. Grenoble Alpes, Inserm, CNRS, Institute for Advanced Biosciences U1209, Grenoble, France
[8] Assistance Publique-Hôpitaux de Paris (AP-HP), Pôle de Biologie Médicale, Paul-Brousse University Hospital, Villejuif, France

- **Figure S1. Bioinformatics workflow implemented to select candidate hepatocyte injury biomarkers**
- **Figure S2. Sequence alignment of human ADH proteins**
- **Figure S3. Evaluation of ADH1B assay performance**
- **Figure S4. Kinetic profiles for ADH1B serum concentrations, ALT activity and INR in ALI/ALF patients**

Figure S1. Bioinformatics workflow implemented to select candidate biomarkers for liver injury.

The workflow diagram below shows each step of the strategy, the tools used, and how they were combined. Box 1: Selection of candidate highly hepatocyte-enriched proteins. Box 2: Elimination of candidate cytoplasmic proteins with transmembrane domains and molecular weight (MW) greater than 85 kDa. Box 3: Retention of candidates without "diseases" information related to liver disease. Box 4: Filtering to retain proteins previously detected by LC–MS/MS in plasma samples, with more than 200 MS/MS observations.

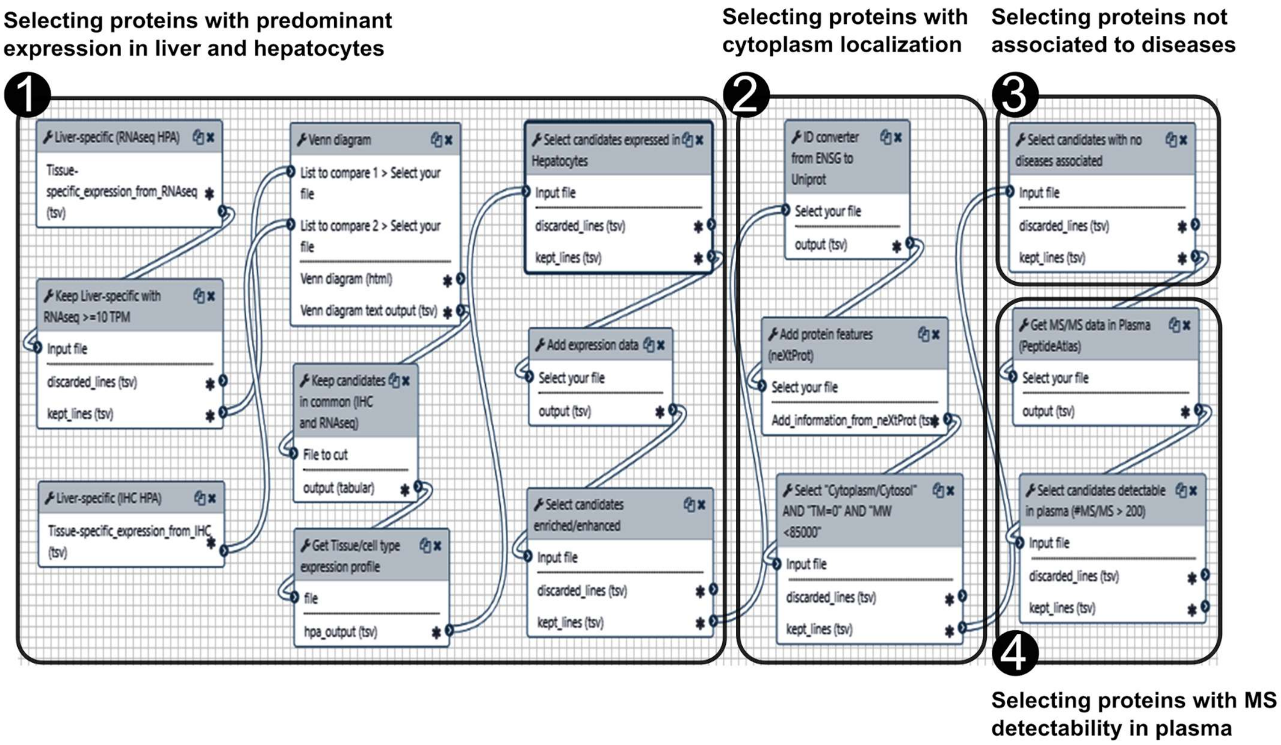

Figure S2. Sequence alignment of human ADH proteins.

The peptides generated by ADH1B proteolysis and monitored using LC-SRM are highlighted in yellow (peptides shared between ADH1 isoforms) or red (peptide AAVLWEVK, specific for ADH1B isoform).

```
SP|P07327|ADH1A_HUMAN ------------MSTAGKVIKCKAAVLWELKKPFSIEEVEVAPPKAHEVRIKMVAVGICG 48
SP|P00325|ADH1B_HUMAN ------------MSTAGKVIKCKAAVLWEVKKPFSIEDVEVAPPKAYEVRIKMVAVGICR 48
SP|P00326|ADH1G_HUMAN ------------MSTAGKVIKCKAAVLWELKKPFSIEEVEVAPPKAHEVRIKMVAAGICR 48
SP|P08319|ADH4_HUMAN  ------------MGTKGKVIKCKAAIAWEAGKPLCIEEVEVAPPKAHEVRIQIIATSLCH 48
SP|P28332|ADH6_HUMAN  ------------MSTTGQVIRCKAAILWKPGAPFSIEEVEVAPPKAKEVRIKVVATGLCG 48
SP|P40394|ADH7_HUMAN  MFAEIQIQDKDRMGTAGKVIKCKAAVLWEQKQPFSIEEIEVAPPKTKEVRIKILATGICR 60
SP|P11766|ADHX_HUMAN  --------------MANEVIKCKAAVAWEAGKPLSIEEIEVAPPKAHEVRIKIIATAVCH 46
                                      .:**:****: *:   *:.**::******: ****:::*..:*

SP|P07327|ADH1A_HUMAN TDDHVVSGT-MVTPLPVILGHEAAGIVESVGEGVTTVKPGDKVIPLAIPQCGKCRICKNP 107
SP|P00325|ADH1B_HUMAN TDDHVVSGN-LVTPLPVILGHEAAGIVESVGEGVTTVKPGDKVIPLFTPQCGKCRVCKNP 107
SP|P00326|ADH1G_HUMAN SDEHVVSGN-LVTPLPVILGHEAAGIVESVGEGVTTVKPGDKVIPLFTPQCGKCRICKNP 107
SP|P08319|ADH4_HUMAN  TDATVIDSKFEGLAFPVIVGHEAAGIVESIGPGVTNVKPGDKVIPLYAPLCRKCKFCLSP 108
SP|P28332|ADH6_HUMAN  TEMKVLGSKHLDLLYPTILGHEGAGIVESIGEGVSTVKPGDKVITLFLPQCGECTSCLNS 108
SP|P40394|ADH7_HUMAN  TDDHVIKGT-MVSKFPVIVGHEATGIVESIGEGVTTVKPGDKVIPLFLPQCRECNACRNP 119
SP|P11766|ADHX_HUMAN  TDAYTLSGADPEGCFPVILGHEGAGIVESVGEGVTKLKAGDTVIPLYIPQCGECKFCLNP 106
                      ::  .:  .       *.*:***.:*****:* **:.:* **.** *  * * :*  * .

SP|P07327|ADH1A_HUMAN ESNYCLKNDVS----NPQGTLQDGTSRFTCRRKPIHHFLGISTFSQYTVVDENAVAKIDA 163
SP|P00325|ADH1B_HUMAN ESNYCLKNDLG----NPRGTLQDGTRRFTCRGKPIHHFLGTSTFSQYTVVDENAVAKIDA 163
SP|P00326|ADH1G_HUMAN ESNYCLKNDLG----NPRGTLQDGTRRFTCSGKPIHHFVGVSTFSQYTVVDENAVAKIDA 163
SP|P08319|ADH4_HUMAN  LTNLCGKISNLKSPASDQQLMEDKTSRFTCKGKPVYHFFGTSTFSQYTVVSDINLAKIDD 168
SP|P28332|ADH6_HUMAN  EGNFCIQFKQS----K-TQLMSDGTSRFTCKGKSIYHFGNTSTFCEYTVIKEISVAKIDA 163
SP|P40394|ADH7_HUMAN  DGNLCIRSDIT----G-RGVLADGTTRFTCKGKPVHHFMNTSTFTEYTVVDESSVAKIDD 174
SP|P11766|ADHX_HUMAN  KTNLCQKIRVT----QGKGLMPDGTSRFTCKGKTILHYMGTSTFSEYTVVADISVAKIDP 162
                        * * :             : * * ****  * : *: . *** :***: :  :****

SP|P07327|ADH1A_HUMAN ASPLEKVCLIGCGFSTGYGSAVNVAKVTPGSTCAVFGLGGVGLSAIMGCKAAGAARIIAV 223
SP|P00325|ADH1B_HUMAN ASPLEKVCLIGCGFSTGYGSAVNVAKVTPGSTCAVFGLGGVGLSAVMGCKAAGAARIIAV 223
SP|P00326|ADH1G_HUMAN ASPLEKVCLIGCGFSTGYGSAVKVAKVTPGSTCAVFGLGGVGLSVVMGCKAAGAARIIAV 223
SP|P08319|ADH4_HUMAN  DANLERVCLLGCGFSTGYGAAINNAKVTPGSTCAVFGLGGVGLSAVMGCKAAGASRIIGI 228
SP|P28332|ADH6_HUMAN  VAPLEKVCLISCGFSTGFGAAINTAKVTPGSTCAVFGLGGVGLSVVMGCKAAGAARIIGV 223
SP|P40394|ADH7_HUMAN  AAPPEKVCLIGCGFSTGYGAAVKTGKVKPGSTCVVFGLGGVGLSVIMGCKSAGASRIIGI 234
SP|P11766|ADHX_HUMAN  LAPLDKVCLLGCGISTGYGAAVNTAKLEPGSVCAVFGLGGVGLAVIMGCKVAGASRIIGV 222
                       :  ::***:.**:***:*:*:: .*: ***.*.*********:.:**** ***:***.:

SP|P07327|ADH1A_HUMAN DINKDKFAKAKELGATECINPQDYKKPIQEVLKEMTDGGVDFSFEVIGRLDTMMASLLCC 283
SP|P00325|ADH1B_HUMAN DINKDKFAKAKELGATECINPQDYKKPIQEVLKEMTDGGVDFSFEVIGRLDTMMASLLCC 283
SP|P00326|ADH1G_HUMAN DINKDKFAKAKELGATECINPQDYKKPIQEVLKEMTDGGVDFSFEVIGRLDTMMASLLCC 283
SP|P08319|ADH4_HUMAN  DINSEKFVKAKALGATDCLNPRDLHKPIQEVIIELTKGGVDFALDCAGGSETMKAALDCT 288
SP|P28332|ADH6_HUMAN  DVNKEKFKKAQELGATECLNPQDLKKPIQEVLFDMTDAGIDFCFEAIGNLDVLAAALASC 283
SP|P40394|ADH7_HUMAN  DLNKDKFEKAMAVGATECISPKDSTKPISEVLSEMTGNNVGYTFEVIGHLETMIDALASC 294
SP|P11766|ADHX_HUMAN  DINKDKFARAKEFGATECINPQDFSKPIQEVLIEMTDGGVDYSFECIGNVKVMRAALEAC 282
                      *:*.:** :*  .***:*:.*:*  ***.**: ::*  .:.: ::  *  ..:  :* .

SP|P07327|ADH1A_HUMAN HEACGTSVIVGVPPDSQNLSMNPMLLLTGRTWKGAILGGFKSKECVPKLVADFMAKKFSL 343
SP|P00325|ADH1B_HUMAN HEACGTSVIVGVPPASQNLSINPMLLLTGRTWKGAVYGGFKSKEGIPKLVADFMAKKFSL 343
SP|P00326|ADH1G_HUMAN HEACGTSVIVGVPPDSQNLSINPMLLLTGRTWKGAIFGGFKSKESVPKLVADFMAKKFSL 343
SP|P08319|ADH4_HUMAN  TAGWGSCTFIGVAAGSKGLTIFPEELIIGRTINGTFFGGWKSVDSIPKLVTDYKNKKFNL 348
SP|P28332|ADH6_HUMAN  NESYGVCVVVGVLPASVQLKISGQLFFSGRSLKGSVFGGWKSRQHIPKLVADYMAEKLNL 343
SP|P40394|ADH7_HUMAN  HMNYGTSVVVGVPPSAKMLTYDPMLLFTGRTWKGCVFGGLKSRDDVPKLVTEFLAKKFDL 354
SP|P11766|ADHX_HUMAN  HKGWGVSVVVGVAASGEEIATRPFQLVTGRTWKGTAFGGWKSVESVPKLVSEYMSKKIKV 342
                          * ...:**   .  :      :. **: :*   ** ** : :****:::  :*:.:

SP|P07327|ADH1A_HUMAN DALITHVLPFEKINEGFDLLHSGKSIRTILMF 375
SP|P00325|ADH1B_HUMAN DALITHVLPFEKINEGFDLLHSGKSIRTVLTF 375
SP|P00326|ADH1G_HUMAN DALITNILPFEKINEGFDLLRSGKSIRTVLTF 375
SP|P08319|ADH4_HUMAN  DALVTHTLPFDKISEAFDLMNQGKSVRTILIF 380
SP|P28332|ADH6_HUMAN  DPLITHTLNLDKINEAVELMKTGKW------- 368
SP|P40394|ADH7_HUMAN  DQLITHVLPFKKISEGFELLNSGQSIRTVLTF 386
SP|P11766|ADHX_HUMAN  DEFVTHNLSFDEINKAFELMHSGKSIRTVVKI 374
                      * ::*: * :.:*.:..:*:. *:
```

Figure S3. Evaluation of ADH1B assay performance.

→ **Determining linearity, accuracy, LLOD, LLOQ and technical precision using a calibration curve**

Six calibration points were created by adding increasing amounts of unlabeled recombinant ADH1B (provided by Abcam) and a fixed amount of ADH1B PSAQ standard to plasma samples (see Table below). ADH1B PSAQ standard is an isotopically-labeled protein analog of ADH1B. The upper limit of the calibration curve (i.e., the concentration of unlabeled protein added) was adjusted in line with expected pathological levels. These levels were estimated from preliminary experiments with two plasma samples from patients with acetaminophen-induced acute liver injury. These two clinical samples were collected at the Clinique Universitaire d'Hépato-gastroentérologie, Centre Hospitalier Universitaire de Grenoble, France, in accordance with ethical guidelines and after obtaining the patient's informed consent. Samples containing only PSAQ standards were also included in the curves. Full-technical replicates of all calibration points were produced (n = 3) to assess technical precision. After spiking, plasma samples were treated according to the established workflow which combines abundant protein removal, digestion with endoLysC/trypsin, and LC-SRM analysis. The ADH1B calibration curve (signature peptide AAVLWEVK) was linear over the concentration range tested. Notably, other peptides generated by ADH1B proteolysis were also monitored, but they were not taken into account for ADH1B quantification in clinical samples as they are shared between several protein isoforms (see Supplementary Figures S2 and S4). The lower limit of detection (LLOD) and the lower limit of quantification (LLOQ) were determined according to the calibration plot method [26].

	Full-technical replicates	Plasma volume (µL)	ADH1B spiked (µg/mL)	ADH1B PSAQ standard spiked (µg/mL)
**Zero sample**	A	14	0	3
	B	14	0	3
	C	14	0	3
**Titration point N°1**	A	14	1	3
	B	14	1	3
	C	14	1	3
**Titration point N°2**	A	14	3	3
	B	14	3	3
	C	14	3	3
**Titration point N°3**	A	14	7	3
	B	14	7	3
	C	14	7	3
**Titration point N°4**	A	14	22	3
	B	14	22	3
	C	14	22	3
**Titration point N°5**	A	14	73	3
	B	14	73	3
	C	14	73	3
**Titration point N°6**	A	14	384	3
	B	14	384	3
	C	14	384	3

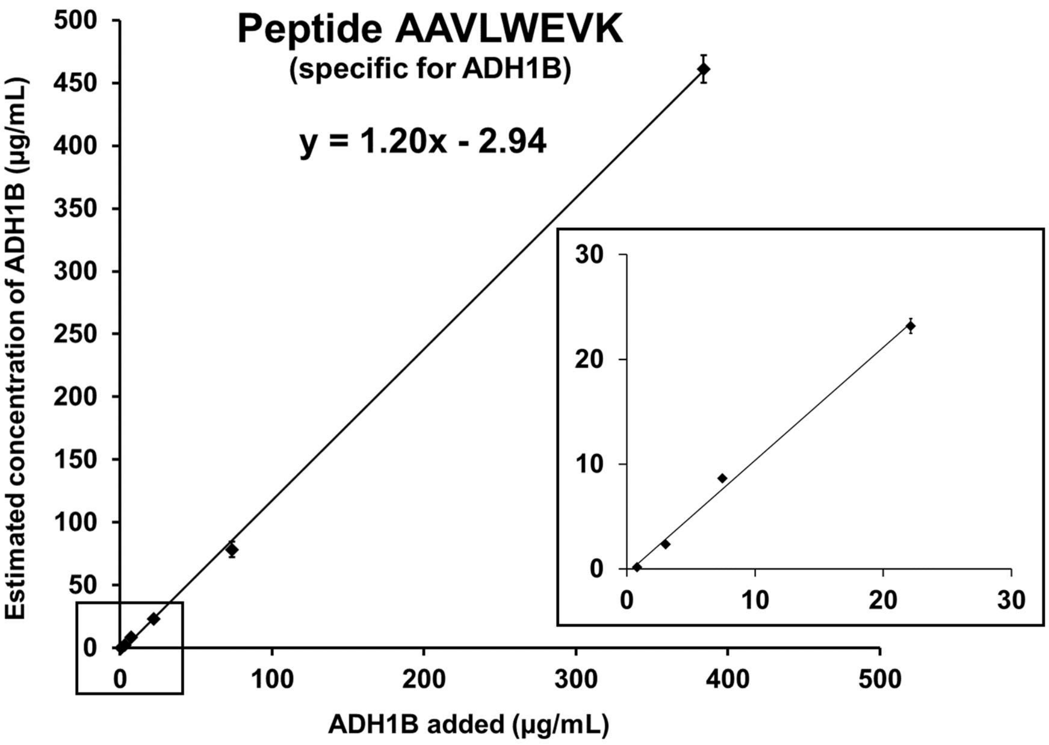

→ **Assessing matrix effects and interferences**

The impact of the blood sampling procedure (plasma or serum fraction) and interfering biological substances (hemoglobin, triglycerides and bilirubin) was assessed in parallel experiments. Various types of samples (n = 2 full-technical replicates per sample) were spiked with defined quantities of ADH1B and its corresponding PSAQ standard. After spiking, samples were submitted to the workflow combining removal of abundant proteins, digestion with endoLysC/trypsin, and LC-SRM analysis. For each sample, the ratio of labeled to unlabeled SRM signals (obtained from the quantifier transition of the AAVLWEVK peptide) were compared to the ratio for the analyte/PSAQ mix digested with endoLysC/trypsin in buffer (50 mM NH_4HCO_3/2 M urea).

**Sample**		**ADH1B**	
		**Measured ratio**	**Bias (%)**
Mix of ADH1B and labeled ADH1B	Replicate 1	0.22	
	Replicate 2	0.20	
	Mean	**0.21**	
Pool of plasma from five healthy donors	Replicate 1	0.18	
	Replicate 2	0.19	
	Mean	**0.19**	**- 10%**
Pool of serum from six healthy donors	Replicate 1	0.18	
	Replicate 2	0.18	
	Mean	**0.18**	**- 14%**
Hemolytic serum (hemoglobin 500 mg/dL)	Replicate 1	0.15	
	Replicate 2	0.17	
	Mean	**0.16**	**- 24%**
Lipemic serum (triglycerides 200 mg/dL)	Replicate 1	0.18	
	Replicate 2	0.17	
	Mean	**0.18**	**- 14%**
Lipemic serum (triglycerides 500 mg/dL)	Replicate 1	0.20	
	Replicate 2	0.21	
	Mean	**0.21**	**0%**
Serum with high bilirubin (total bilirubin 326 μmol/L)	Replicate 1	0.19	
	Replicate 2	0.19	
	Mean	**0.19**	**- 10%**
Serum with medium bilirubin (total bilirubin 82 μmol/L)	Replicate 1	0.18	
	Replicate 2	0.18	
	Mean	**0.18**	**- 14%**

→ Assessing analyte stability

Two serum samples from ALI patients were processed either immediately or after 3 months of storage at -80 °C. Labeled ADH1B (3 µg/mL) was spiked just before sample processing. Endogenous concentrations of ADH1B were estimated in each sample and the difference (%) between the initial and delayed measures was calculated (see Table below).

**Sample**		**ADH1B concentration**
Clinical sample 1	T0	9.87 µg/mL
	T0 + 3 months	12.42 µg/mL
	**Bias**	**25.8 %**
Clinical sample 2	T0	4.59 µg/mL
	T0 + 3 months	5.22 µg/mL
	**Bias**	**13.7 %**
**Mean biais**		**19.8 %**

→ Assessing sample stability during handling

A "disease" pool was created by mixing equal volumes of 20 serum samples from ALI patients. This pooled sample was divided into aliquots (14 µL each) to which labeled ADH1B (3 µg/mL) was added before removing abundant proteins and digesting the proteins with endoLysC/trypsin. Each digest was desalted, dried by vacuum centrifugation and resuspended in 15 µL of 2% acetonitrile/0.1% formic acid. LC-SRM analyses were performed using 6 µL of each digest, as follows:

- immediately (two replicates),
- after sample storage at room temperature (21 °C) for 4 h (two replicates),
- after storage at 4 °C for 24 h (two replicates),
- after two freeze-thaw cycles (two replicates),
- after frozen storage for 30 days at -20 °C (two replicates).

Endogenous concentrations of ADH1B were estimated in the different technical replicates and the difference (%) between each stressed storage condition and immediate analysis was evaluated (see Table below).

**Type of sample and analysis**		**ADH1B concentration**
Processed "disease" pool<br>Direct analysis after sample processing	Replicate 1	22.8 µg/mL
	Replicate 2	21.8 µg/mL
	Mean concentration	**22.3 µg/mL**
Processed "disease" pool<br>Analysis after 4 h at room temperature	Replicate 1	21.5 µg/mL
	Replicate 2	22.1 µg/mL
	Mean concentration	**21.8 µg/mL**
	**Bias**	**-2.2%**
Processed "disease" pool<br>Analysis after 24 h at 4 °C	Replicate 1	22.9 µg/mL
	Replicate 2	22.4 µg/mL
	Mean concentration	**22.7 µg/mL**
	**Bias**	**1.8%**
Processed "disease" pool<br>Analysis after two freeze-thaw cycles	Replicate 1	22.2 µg/mL
	Replicate 2	22.5 µg/mL
	Mean concentration	**22.4 µg/mL**
	**Bias**	**1.0%**
Processed "disease" pool<br>Analysis after frozen storage (-20 °C) for 30 days	Replicate 1	23.7 µg/mL
	Replicate 2	21.5 µg/mL
	Mean concentration	**22.6 µg/mL**
	**Bias**	**1.4%**

→ Assessing peptide stability (post-extraction stability)

Aliquots of the "disease" pool (14 µL each) were spiked with labeled ADH1B (3 µg/mL) before removing abundant proteins and digesting with endoLysC/trypsin. Then, each digest was spiked with a doubly-labeled peptide differing in mass from the one generated by labeled ADH1B standard ([$^{13}C_6$, $^{15}N_2$] L-lysine, [$^{13}C_5$, ^{15}N] L-valine labeled peptide AAVLWEVK, HeavyPeptide AQUA Grade Ultimate, ThermoFischer, spiked at 5.6 femtomoles/µL). After desalting, digests were dried by vacuum centrifugation and resuspended in 15 µL of 2% acetonitrile/0.1% formic acid. LC-SRM analyses were performed using 6 µL of each digest. The doubly-labeled peptide was

used as a standard to quantify and determine the stability of the endogenous version under several stressed conditions (n = 3 full-technical replicates per condition):

– storage at room temperature (21 °C) for 4 h,
– storage at 4 °C for 24 h,
– two freeze-thaw cycles,
– frozen storage for > 30 days at -80 °C.

**Type of sample and analysis**		**Peptide AAVLWEVK concentration**
Processed "disease" pool<br>Direct analysis of the peptide digest	Replicate 1	2.8 femtomoles/µL
	Replicate 2	2.8 femtomoles/µL
	Replicate 3	2.6 femtomoles/µL
	Mean concentration	**2.7 femtomoles/µL**
Processed "disease" pool<br>Analysis after storing the peptide digest for 4 h at room temperature	Replicate 1	2.8 femtomoles/µL
	Replicate 2	2.7 femtomoles/µL
	Replicate 3	3.0 femtomoles/µL
	Mean concentration	**2.8 femtomoles/µL**
	**Bias**	**3.7%**
Processed "disease" pool<br>Analysis after storing the peptide digest for 24 h at 4 °C (autosampler stability)	Replicate 1	2.9 femtomoles/µL
	Replicate 2	2.7 femtomoles/µL
	Replicate 3	2.8 femtomoles/µL
	Mean concentration	**2.8 femtomoles/µL**
	**Bias**	**3.7%**
Processed "disease" pool<br>Peptide digest analysis after two freeze-thaw cycles	Replicate 1	2.8 femtomoles/µL
	Replicate 2	2.6 femtomoles/µL
	Replicate 3	2.7 femtomoles/µL
	Mean concentration	**2.7 femtomoles/µL**
	**Bias**	**0.0%**
Processed "disease" pool<br>Peptide digest analysis after frozen storage (-80 °C) for > 30 days	Replicate 1	3.0 femtomoles/µL
	Replicate 2	2.6 femtomoles/µL
	Replicate 3	3.0 femtomoles/µL
	Mean concentration	**2.9 femtomoles/µL**
	**Bias**	**7.4%**

→ Evaluation of repeatability and reproducibility

The "disease" pool was spiked with 3 µg/mL of labeled ADH1B and was splitted into several aliquots (14 µL each). Each aliquot was submitted to the workflow combining removal of abundant proteins, digestion with endoLysC/trypsin, and LC-SRM

analysis. The endogenous concentrations of ADH1B were estimated in five technical replicates of the disease pool sample analyzed in the same day and five technical replicates analyzed over five successive days.

Intraday analytical variability (repeatability)

Analyte	Replicate 1	Replicate 2	Replicate 3	Replicate 4	Replicate 5	Mean concentration	CV
**ADH1B**	23.3 μg/mL	21.6 μg/mL	22.3 μg/mL	23.1 μg/mL	22.1 μg/mL	**22.5 μg/mL**	**3.2%**

Interday analytical variability (reproducibility)

Analyte	Replicate 1	Replicate 2	Replicate 3	Replicate 4	Replicate 5	Mean concentration	CV
**ADH1B**	23.3 μg/mL	23.5 μg/mL	21.6 μg/mL	23.3 μg/mL	21.7 μg/mL	**22.7 μg/mL**	**4.2%**

Figure S4. Kinetic profiles for ADH1B serum concentrations, ALT activity and INR in ALI/ALF patients.

Serum concentrations of ADH1B were determined by LC-SRM as described in the ***Materials and Methods*** section. For each patient, the time-course for ADH1B levels was compared to that for the biological parameters used in routine clinical practice to monitor ALI/ALF, *i.e.*, ALT activity and INR (reported as an indicator of coagulation defects). As complementary information, ADH1 isoform quantities estimated from the shared peptides INEGFDLLHSGK and FSLDALITHVLPFEK (for ADH1A and ADH1B) and IDAASPLEK (for ADH1A, ADH1B and ADH1C) are also reported on the kinetic profiles. For each patient and time-point, all quantification values are presented in Supplementary Table S4.

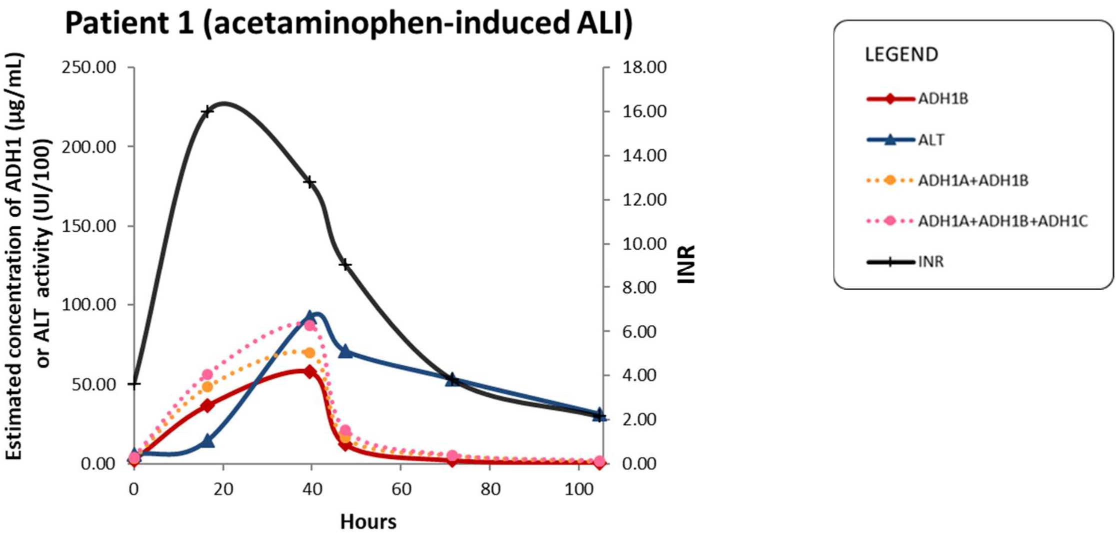

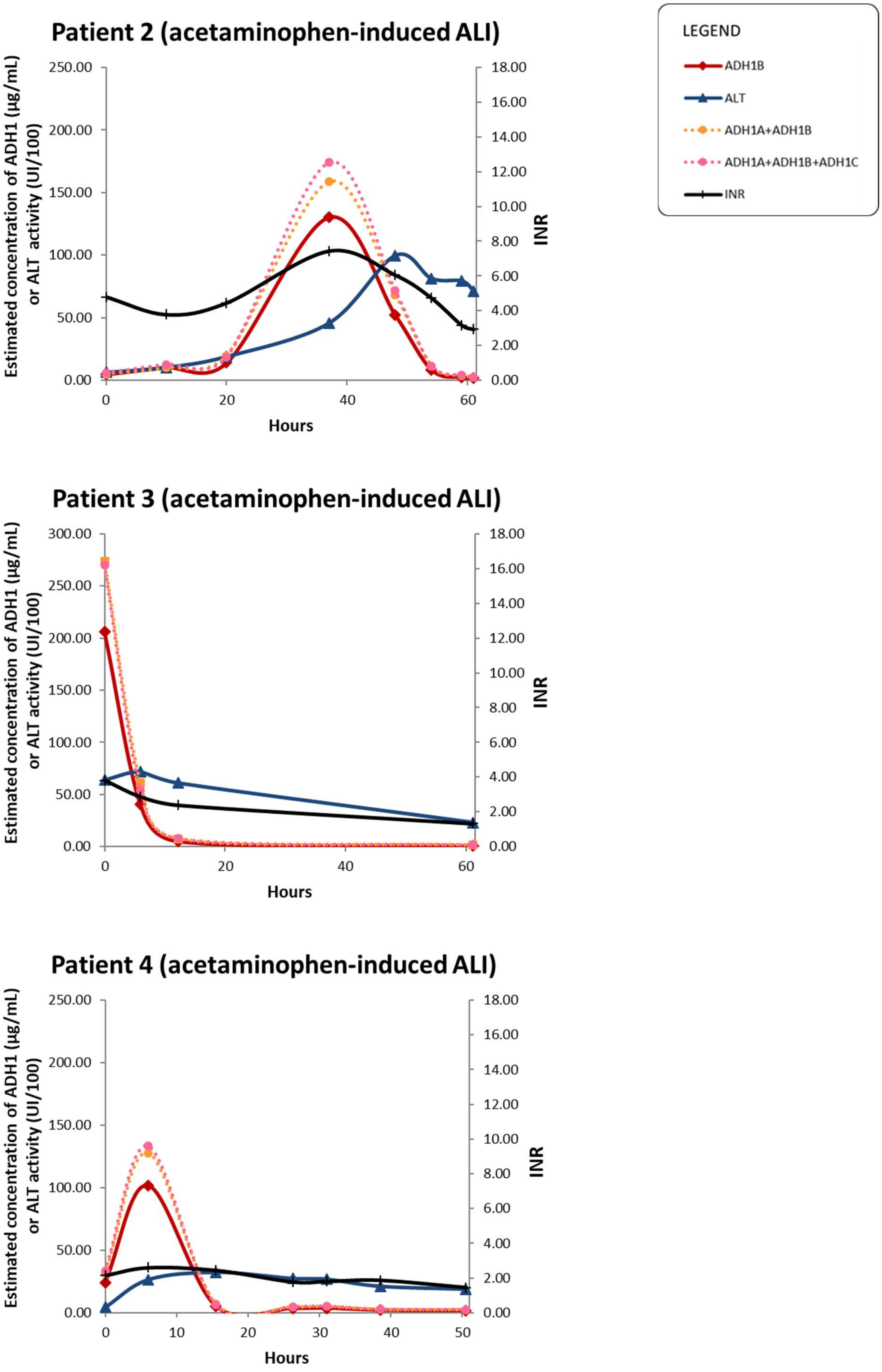
Patient 2 (acetaminophen-induced ALI)
Estimated concentration of ADH1 (µg/mL) or ALT activity (UI/100)
INR
Hours
Patient 3 (acetaminophen-induced ALI)
Estimated concentration of ADH1 (µg/mL) or ALT activity (UI/100)
INR
Hours
Patient 4 (acetaminophen-induced ALI)
Estimated concentration of ADH1 (µg/mL) or ALT activity (UI/100)
INR
Hours
LEGEND
ADH1B
ALT
ADH1A+ADH1B
ADH1A+ADH1B+ADH1C
INR

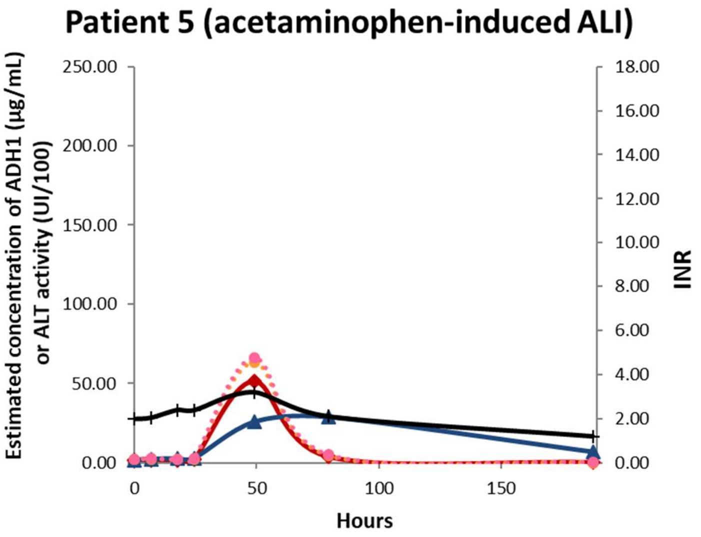
Patient 5 (acetaminophen-induced ALI)
Estimated concentration of ADH1 (µg/mL) or ALT activity (UI/100)
INR
Hours
250.00
200.00
150.00
100.00
50.00
0.00
18.00
16.00
14.00
12.00
10.00
8.00
6.00
4.00
2.00
0.00
0
50
100
150

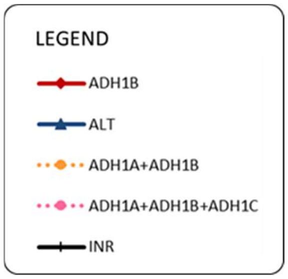
LEGEND
ADH1B
ALT
ADH1A+ADH1B
ADH1A+ADH1B+ADH1C
INR

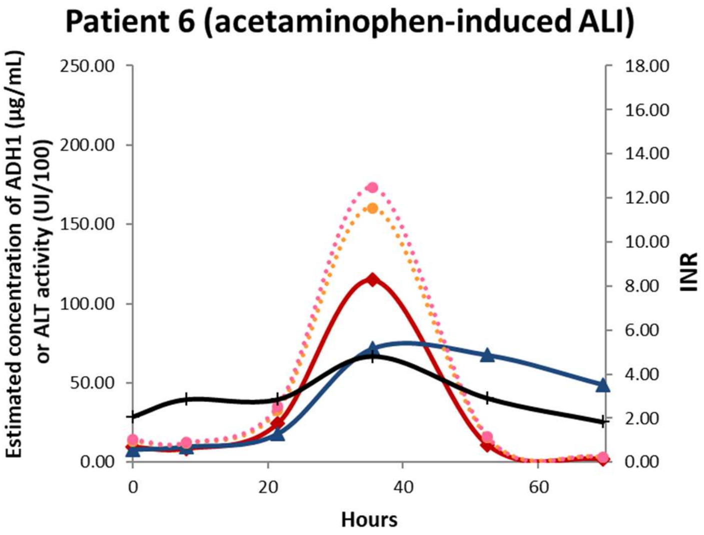
Patient 6 (acetaminophen-induced ALI)
Estimated concentration of ADH1 (µg/mL) or ALT activity (UI/100)
INR
Hours
250.00
200.00
150.00
100.00
50.00
0.00
18.00
16.00
14.00
12.00
10.00
8.00
6.00
4.00
2.00
0.00
0
20
40
60

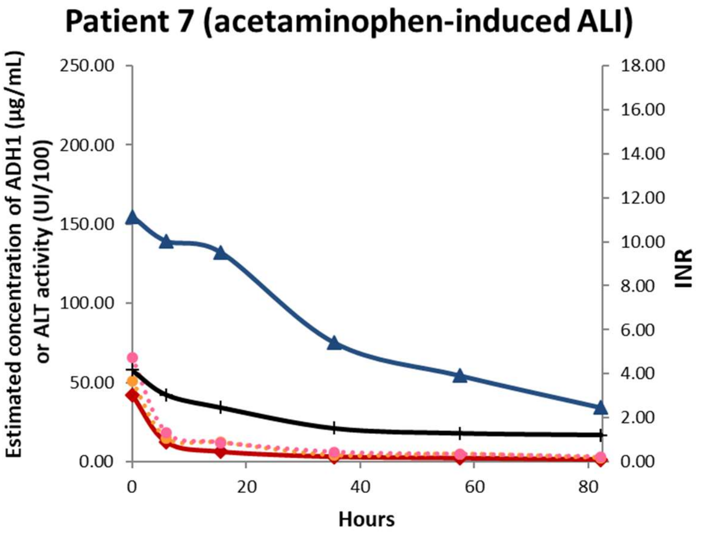
Patient 7 (acetaminophen-induced ALI)
Estimated concentration of ADH1 (µg/mL) or ALT activity (UI/100)
INR
Hours
250.00
200.00
150.00
100.00
50.00
0.00
18.00
16.00
14.00
12.00
10.00
8.00
6.00
4.00
2.00
0.00
0
20
40
60
80

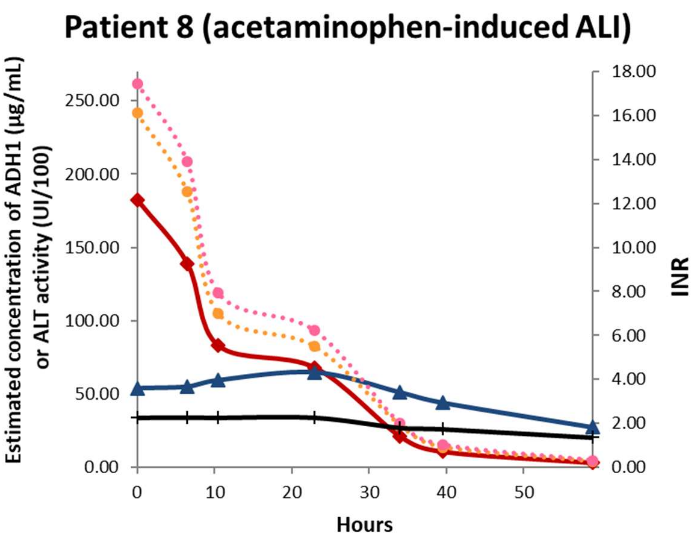
Patient 8 (acetaminophen-induced ALI)
Estimated concentration of ADH1 (µg/mL) or ALT activity (UI/100)
INR
Hours
0.00
50.00
100.00
150.00
200.00
250.00
2.00
4.00
6.00
8.00
10.00
12.00
14.00
16.00
18.00
0
10
20
30
40
50

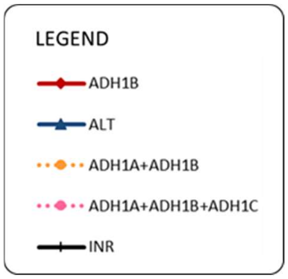
LEGEND
ADH1B
ALT
ADH1A+ADH1B
ADH1A+ADH1B+ADH1C
INR

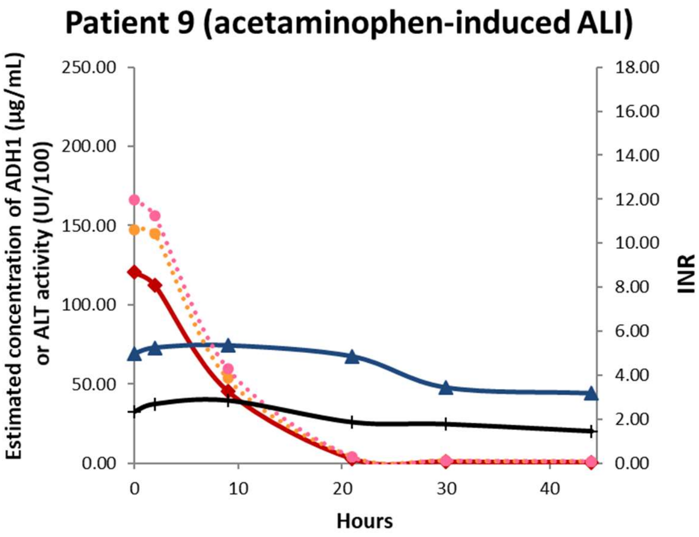
Patient 9 (acetaminophen-induced ALI)
Estimated concentration of ADH1 (µg/mL) or ALT activity (UI/100)
INR
Hours
0.00
50.00
100.00
150.00
200.00
250.00
2.00
4.00
6.00
8.00
10.00
12.00
14.00
16.00
18.00
0
10
20
30
40

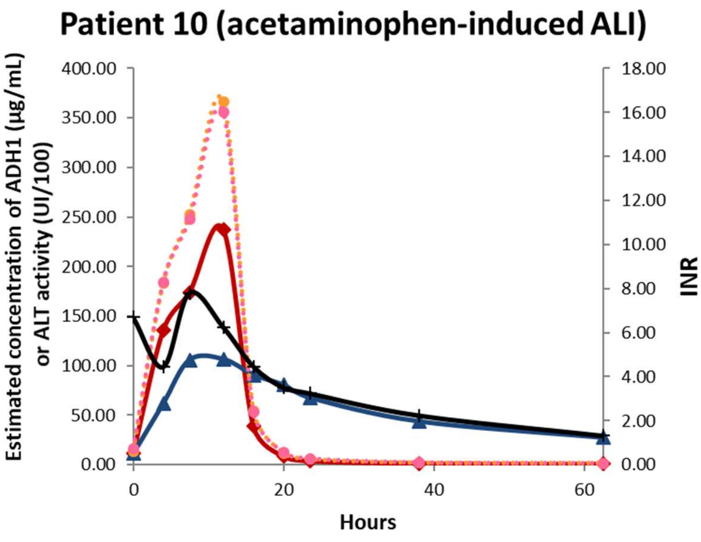
Patient 10 (acetaminophen-induced ALI)
Estimated concentration of ADH1 (µg/mL) or ALT activity (UI/100)
INR
Hours
0.00
50.00
100.00
150.00
200.00
250.00
300.00
350.00
400.00
2.00
4.00
6.00
8.00
10.00
12.00
14.00
16.00
18.00
0
20
40
60

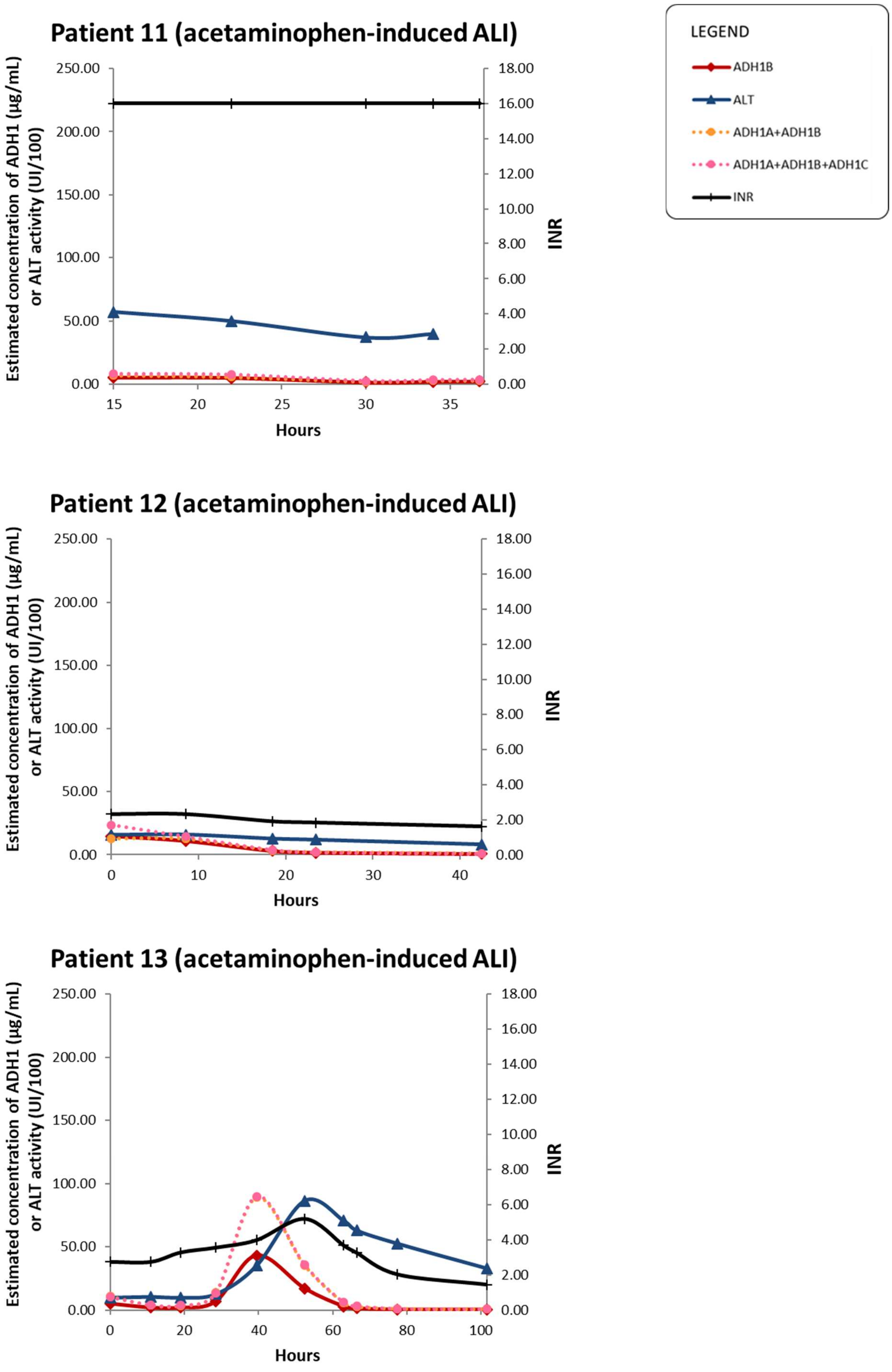

Patient 11 (acetaminophen-induced ALI)
Patient 12 (acetaminophen-induced ALI)
Patient 13 (acetaminophen-induced ALI)
Estimated concentration of ADH1 (µg/mL) or ALT activity (UI/100)
INR
Hours
0.00
50.00
100.00
150.00
200.00
250.00
2.00
4.00
6.00
8.00
10.00
12.00
14.00
16.00
18.00
15
20
25
30
35
0
10
40
60
80
100
LEGEND
ADH1B
ALT
ADH1A+ADH1B
ADH1A+ADH1B+ADH1C
INR

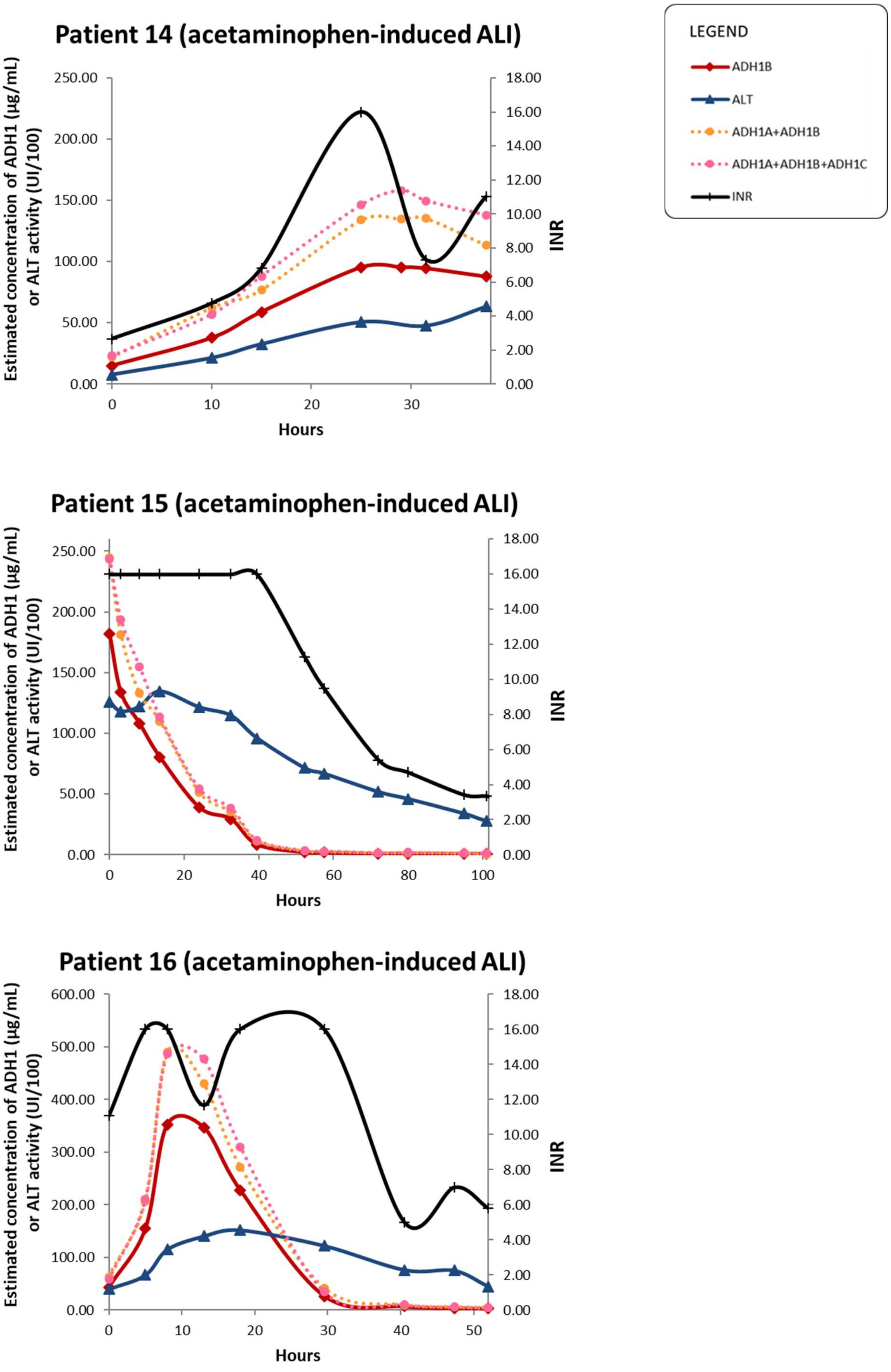
Patient 14 (acetaminophen-induced ALI)
Patient 15 (acetaminophen-induced ALI)
Patient 16 (acetaminophen-induced ALI)
Estimated concentration of ADH1 (µg/mL) or ALT activity (UI/100)
INR
Hours
LEGEND
ADH1B
ALT
ADH1A+ADH1B
ADH1A+ADH1B+ADH1C
INR

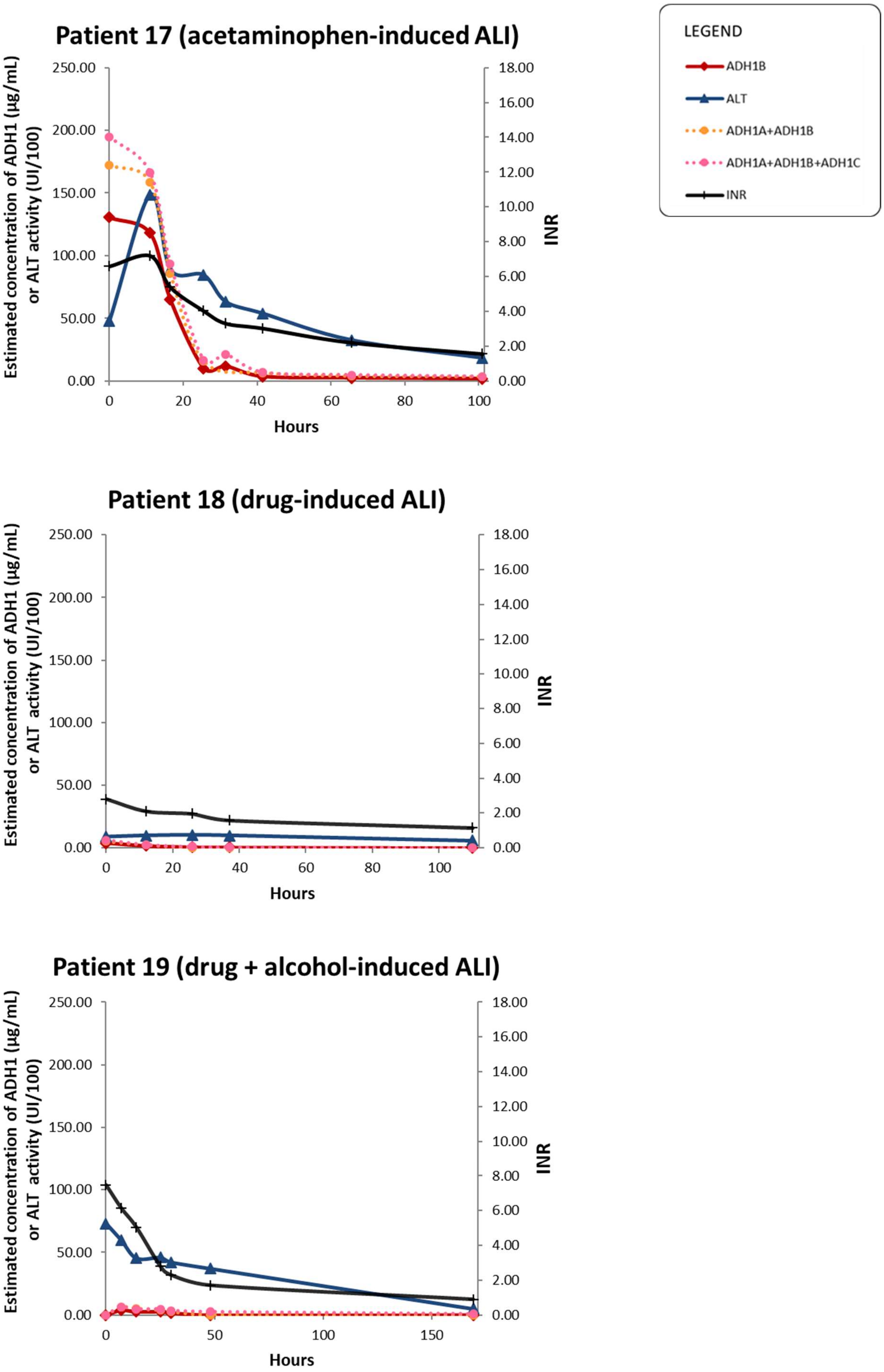
Patient 17 (acetaminophen-induced ALI)
Patient 18 (drug-induced ALI)
Patient 19 (drug + alcohol-induced ALI)
Estimated concentration of ADH1 (µg/mL) or ALT activity (UI/100)
INR
Hours
LEGEND
ADH1B
ALT
ADH1A+ADH1B
ADH1A+ADH1B+ADH1C
INR

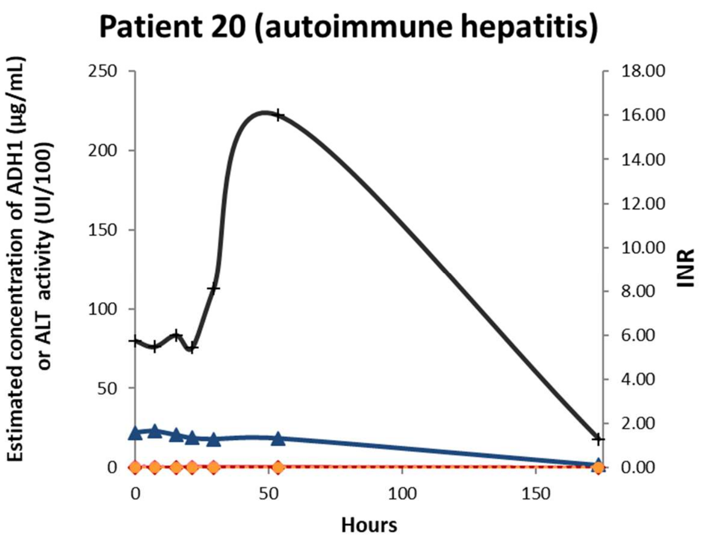
Patient 20 (autoimmune hepatitis)
Estimated concentration of ADH1 (µg/mL) or ALT activity (UI/100)
250
200
150
100
50
0
18.00
16.00
14.00
12.00
10.00
8.00
6.00
4.00
2.00
0.00
INR
0
50
100
150
Hours

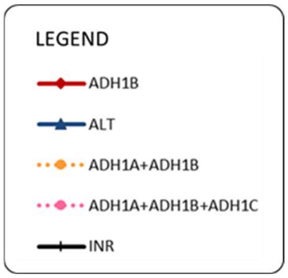
LEGEND
ADH1B
ALT
ADH1A+ADH1B
ADH1A+ADH1B+ADH1C
INR

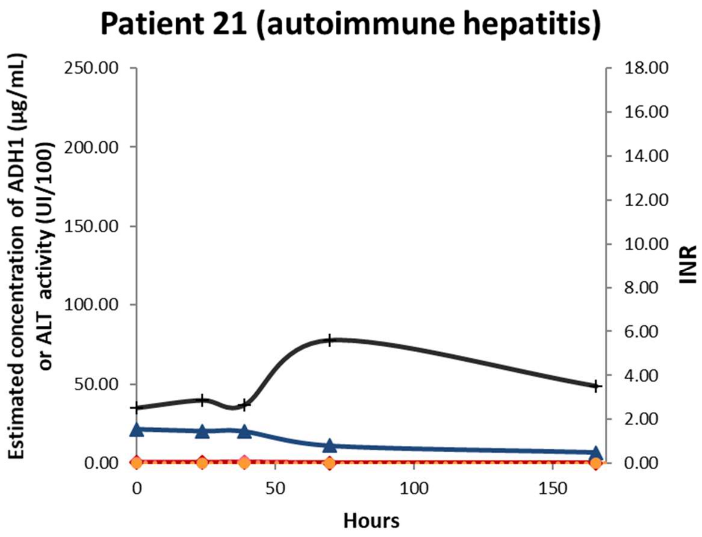
Patient 21 (autoimmune hepatitis)
Estimated concentration of ADH1 (µg/mL) or ALT activity (UI/100)
250.00
200.00
150.00
100.00
50.00
0.00
18.00
16.00
14.00
12.00
10.00
8.00
6.00
4.00
2.00
0.00
INR
0
50
100
150
Hours

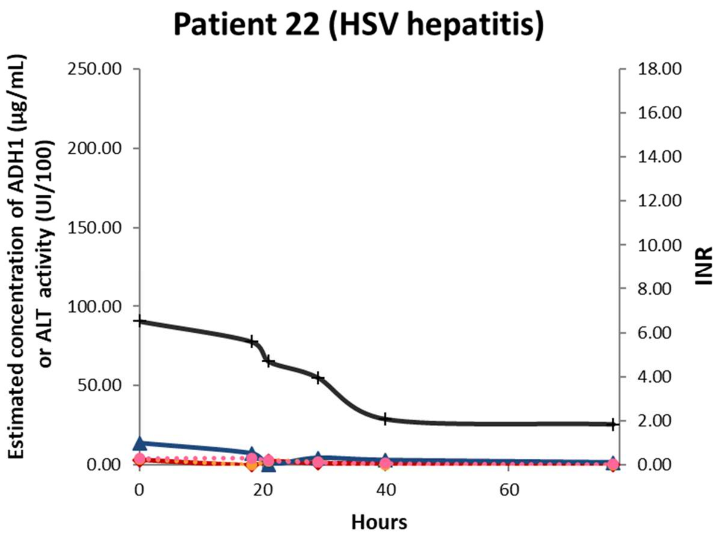
Patient 22 (HSV hepatitis)
Estimated concentration of ADH1 (µg/mL) or ALT activity (UI/100)
250.00
200.00
150.00
100.00
50.00
0.00
18.00
16.00
14.00
12.00
10.00
8.00
6.00
4.00
2.00
0.00
INR
0
20
40
60
Hours
